# Extensive intratumor regional epigenetic heterogeneity in clear cell renal cell carcinoma targets kidney enhancers and is associated with poor outcome

**DOI:** 10.1186/s13148-023-01471-3

**Published:** 2023-04-29

**Authors:** Louis Y. El Khoury, Xiaoyu Pan, Ryan A. Hlady, Ryan T. Wagner, Shafiq Shaikh, Liguo Wang, Mitchell R. Humphreys, Erik P. Castle, Melissa L. Stanton, Thai H. Ho, Keith D. Robertson

**Affiliations:** 1 Department of Molecular Pharmacology and Experimental Therapeutics, Mayo Clinic, Rochester, MN USA; 2 Department of Biochemistry and Molecular Biology, Mayo Clinic, Rochester, MN USA; 3 Division of Biomedical Statistics and Informatics, Department of Health Science Research, Mayo Clinic, Rochester, MN USA; 4 Department of Urology, Mayo Clinic Arizona, Phoenix, AZ USA; 5 Department of Urology, Tulane University School of Medicine, New Orleans, LA USA; 6 Department of Laboratory Medicine and Pathology, Mayo Clinic, Phoenix, AZ USA; 7 Division of Hematology and Medical Oncology, Mayo Clinic Arizona, Scottsdale, AZ USA

**Keywords:** Clear cell renal cell cancer, Intra-tumor heterogeneity, DNA methylation, 5mC, Epigenomics, CNV, ccRCC, SETD2, H3K36me3, Metastasis

## Abstract

**Background:**

Clear cell renal cell cancer (ccRCC), the 8th leading cause of cancer-related death in the US, is challenging to treat due to high level intratumoral heterogeneity (ITH) and the paucity of druggable driver mutations. CcRCC is unusual for its high frequency of epigenetic regulator mutations, such as the *SETD2* histone H3 lysine 36 trimethylase (H3K36me3), and low frequency of traditional cancer driver mutations. In this work, we examined epigenetic level ITH and defined its relationships with pathologic features, aspects of tumor biology, and *SETD2* mutations.

**Results:**

A multi-region sampling approach coupled with EPIC DNA methylation arrays was conducted on a cohort of normal kidney and ccRCC. ITH was assessed using DNA methylation (5mC) and CNV-based entropy and Euclidian distances. We found elevated 5mC heterogeneity and entropy in ccRCC relative to normal kidney. Variable CpGs are highly enriched in enhancer regions. Using intra-class correlation coefficient analysis, we identified CpGs that segregate tumor regions according to clinical phenotypes related to tumor aggressiveness. *SETD2* wild-type tumors overall possess greater 5mC and copy number ITH than *SETD2* mutant tumor regions, suggesting SETD2 loss contributes to a distinct epigenome. Finally, coupling our regional data with TCGA, we identified a 5mC signature that links regions within a primary tumor with metastatic potential.

**Conclusion:**

Taken together, our results reveal marked levels of epigenetic ITH in ccRCC that are linked to clinically relevant tumor phenotypes and could translate into novel epigenetic biomarkers.

**Supplementary Information:**

The online version contains supplementary material available at 10.1186/s13148-023-01471-3.

## Background

Clear cell renal cell carcinoma (ccRCC) accounts for ~ 75% of kidney cancers and is the 8th leading cause of cancer death in the USA. It is also well established as a tumor with a high degree of genetic intratumor heterogeneity (ITH) [1]. Despite intensive surgical efforts, approximately 30% of ccRCC patients experience metastatic progression, typically within three years of surgery. Although adjuvant immunotherapy has been approved, the data for overall survival benefit are not mature [2]. Completion of The Cancer Genome Atlas (TCGA) Project enabled identification of actionable mutations in virtually every solid tumor, and those mutational profiles now drive clinical treatment decisions [3]. One major exception, however, is RCC, where the current standard of care, immune checkpoint inhibitor and anti-VEGF therapy, does not take into account chromatin regulator mutations or ITH [4–6]. After first-line therapy, response rates are 20%, highlighting the need to identify novel drivers and biomarkers to advance individualized treatment of ccRCC patients [5, 7].

The importance of intratumor heterogeneity (ITH), driven by genetic, epigenetic, transcriptional, and tumor microenvironment differences, to cancer biology and clinical outcome has been recognized for some time [8]. CcRCC is characterized by high-level ITH at the gene mutation, copy number variation (CNV), and treatment response levels [1, 9, 10]. For example, only 34% of all mutations detected by a multi-region sequencing approach were present in all regions of the tumor; of known ccRCC driver genes, only *VHL* was mutated ubiquitously [11]. Mutational ITH was also observed among epigenetic regulators, including *SETD2* and *KDM5C*, which sustained multiple unique and spatially distinct inactivating mutations within individual tumor regions. That these subclonal events are important is evident given that their influence on a common truncal event (e.g., VHL loss) leads to diverse clinical outcomes [1, 11]. Reconstructing tumor clonal architectures and identifying common aberrations located at the trunks of phylogenetic trees are expected to lead to more robust biomarkers and novel therapeutic approaches. In the case of ccRCC, however, *VHL* is the only consistent truncal mutation, which has only recently become targetable through its downstream HIF effectors, demonstrating the need to delve deeper into other processes contributing to ITH [1, 11, 12]. ITH, combined with the polygenic nature of drug resistance, likely contributes to the failure of most targeted therapies, including immune therapies that are the mainstay of ccRCC treatment [4–6].

The contribution of ITH to tumor evolution, metastasis, and treatment failure also arises through epigenetic mechanisms, involving heritable changes in gene expression driven by remodeling of epigenetic marks at the DNA and histone levels. Several studies have linked DNA methylation (5mC) aberrations, hypermethylation events in particular, to poor outcome and mutation of the *SETD2* histone H3 lysine 36 trimethylase (H3K36me3) in ccRCC [13–15]. Epigenetic mechanisms play an important role in driving ITH given their functions in modulating expression and responding to environmental cues [16]. Indeed, phenotypic plasticity involving differentiation state-related epigenetic programs has been reported as a key driver of drug resistance through heritable changes in expression programs, independent of genetic aberrations [17]. Consistent with this notion, variance in cell transcriptional programs across tumor types is often independent of genetic-level ITH [18], but is linked to variability in 5mC patterns in genetically homogeneous cell populations [19]. 5mC patterns positively promote expression when localized to gene bodies or repress transcription when present in promoters and enhancers [20]. 5mC also varies between cell types, individuals, and with age, thus providing a rich substrate on which cancer cell properties such as inflammation, metastasis, and drug resistance may be selected for by Darwinian evolutionary forces [21]. CcRCC metastases exhibit few novel de novo mutations, suggesting that most of the diversity promoting metastatic dissemination accumulates in the primary tumor and/or that yet-to-be discovered epigenetic alterations drive selection of metastasis-competent cell populations [22].

In the present study, we sought to gain a better understanding of epigenetic ITH at the level of methylation within ccRCC by integrating 5mC heterogeneity with genetic and pathologic data, determine the specific influence of *SETD2* mutations on ITH given its role in regulating DNA methylation, and apply evolutionary principles to discover novel epigenetic cancer driver events. To accomplish this, we sampled spatially distinct regions from twelve genotyped ccRCC patients and three normal kidneys and performed genome-wide 5mC and copy number analysis using the MethylationEPIC BeadChip (850 k) array. Results were further correlated with immunohistochemical quantification of the same regions for 5mC, H3K36me3 (the mark catalyzed by SETD2), and other pathologic measures. We observed marked heterogeneity at the pathologic level including both H3K36me3 and 5mC marks. While epigenetic ITH in normal kidney was minimal and localized primarily to gene bodies, epigenetic ITH in ccRCC was dramatically elevated and highly enriched in kidney enhancers. CcRCC also displayed markedly elevated methylation-level entropy, which was significantly higher in *SETD2* wt tumors and correlated with markers of tumor aggressiveness. Phyloepigenetic analysis revealed novel epigenetically deregulated drivers and that methylation contributed more to diversity within a tumor than CNV level genetic ITH. Finally, by interfacing our epigenetic ITH data with TCGA data, we uncover a novel 5mC signature of metastasis that may be useful for assessing metastatic potential. Overall, *SETD2* wt/H3K36me3-positive tumor regions showed more epigenetic and genetic level ITH, suggesting that *SETD2* mutations drive an epigenetic landscape that promotes a distinct and more homogeneous epigenome associated with adverse outcomes.

## Results

### Patient and tumor characteristics, and evidence for widespread pathologic-level ITH in ccRCC

**Fig. 1:**
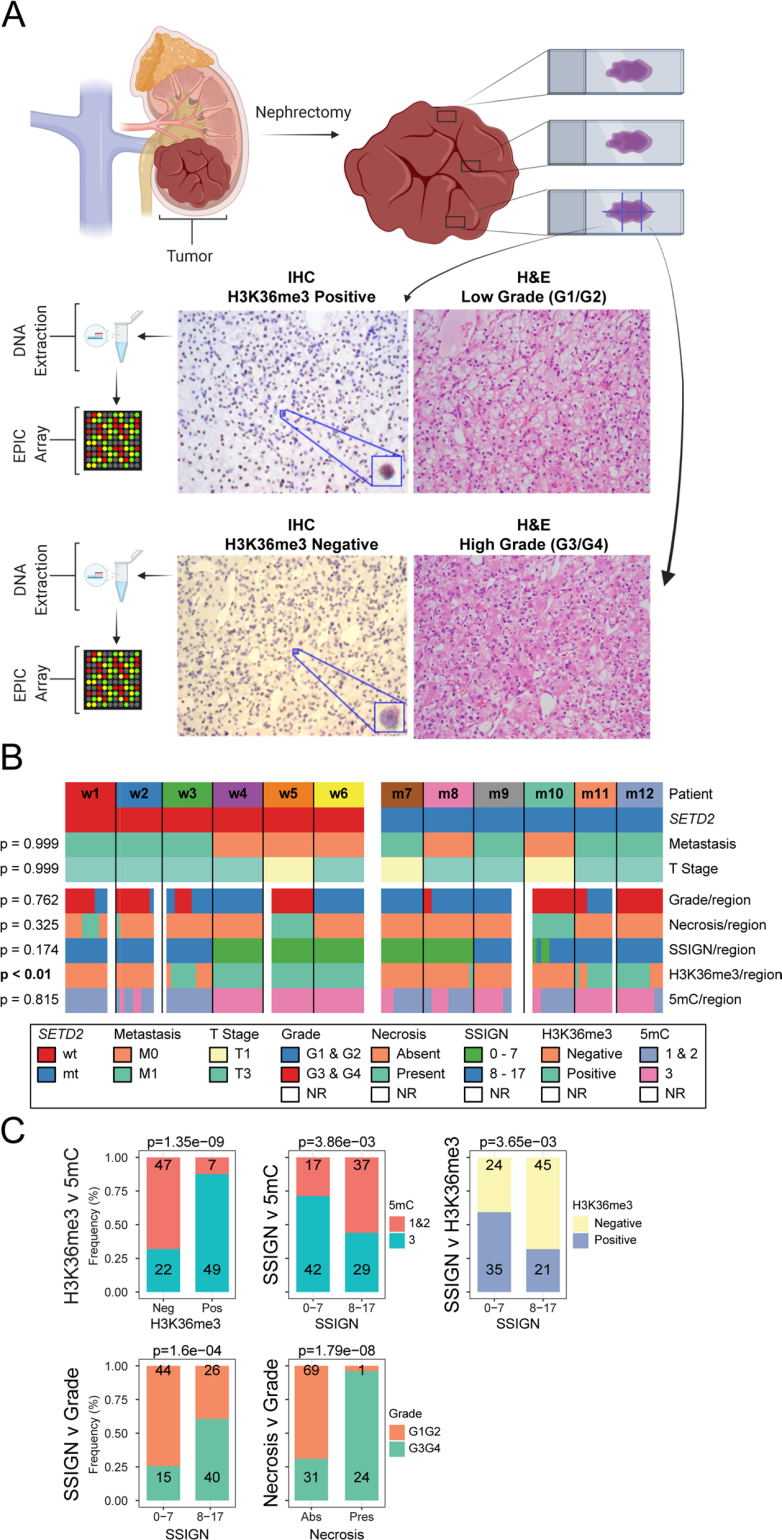
Strategy for defining intratumoral heterogeneity and summary of pathology-level heterogeneity within our ccRCC cohort.** A** Summary of sample acquisition methodology to profile 5mC in distinct tumor regions. Multiple regions of each resected tumor are fixed in FFPE blocks, sectioned, and embedded on microscopic slides. Sequential slides from each block are divided into sections that are assessed independently for H&E, IHC, and 5mC. In panel A (bottom), we illustrate pathology-level ITH represented as different nuclear grade and H3K36me3 levels between neighboring regions of the same tumor. DNA is extracted from these sections independently for downstream analysis. Figure created with Biorender.com **B** Oncoprint showing patient-level results (*SETD2* mutational status, metastasis, and T stage) of all 12 ccRCCs, and below that region-specific results (nuclear grade, necrosis, SSIGN score, H3K36me3 IHC status, and 5mC IHC status). Tumors are grouped according to their *SETD2* mutational status (wt to the left and mt to the right). P-values are the outcome of *χ*^2^ comparison of each criterion between *SETD2* wt and mt tumors. Some tumor regions could not be scored (NR, white blocks). **C** Barplots showing a significant pairwise association of 5mC with H3K36me3 (Neg = negative, Pos = positive) and SSIGN score, H3K36me3 with SSIGN score, and nuclear grade with necrosis (P = present, A = absent) and SSIGN score. Frequencies are plotted on the y-axis, and count is indicated in each section of the colored bars

We identified ccRCCs from our tissue repository containing multiple distinct regions of the tumor (Additional file 1: Table S1). From formaldehyde fixed paraffin embedded (FFPE) samples, tumor material was sectioned, mounted onto microscope slides, and then, further sub-divided into sections that were isolated for genomic DNA preparation, as shown schematically in Fig. 1A (top). This resulted in 10–13 distinct regions from 2–5 blocks per patient (138 sections in total, Additional file 1: Table S2). In parallel, each region was scored by H&E pathologic analysis of key tumor features such as grade, stage, and necrosis, and IHC was used to assess levels of H3K36me3 (the mark written by the SETD2 histone methyltransferase), and DNA methylation (5mC). Thus, each distinct region within a tumor underwent both microscopic assessment of clinical pathology and molecular assessment of DNA methylation.

A single region from each tumor representing the highest nuclear grade was analyzed for gene mutations using an established 600 cancer gene hybridization-capture approach in a CLIA-certified laboratory to a median sequencing depth of at least 650x. Analysis was performed to identify base substitutions, insertions/deletions, gene fusions, rearrangements, and copy number variation (CNV) relative to a normal control kidney sample [23]. A summary of the mutations identified in each tumor sample is summarized in Additional file 1: Table S3. As such, some data presented are on a per patient basis (e.g., *SETD2* mutation status, presence of metastasis) while other data are presented on per region basis (e.g., H3K36me3 status). The tumors were chosen to comprise an equal mix of *SETD2* wild-type (wt) and *SETD2* mutant (mt) given connections that we and others have reported linking *SETD2* mutation to poor outcome and regulation of 5mC [24–27]. Analysis of sequencing data revealed that genes commonly mutated in ccRCC, including *VHL* (10/12 patients) and *PBRM1* (8/12 patients), are the two most frequently mutated genes, indicating that our ccRCC samples have a mutational profile consistent with other large tumor genome sequencing studies such as TCGA KIRC [14] (Additional file 1: Table S3). The cohort also contained a comparable representation of ccRCCs that underwent metastasis vs those that did not (7/12 patients, Table 1). For one case, we also obtained and analyzed a single region from a matched synchronous pancreatic metastasis (Table 1).

**Table 1:** Summary of patient clinical and pathologic information

	Normal (*n* = 3)	All Tumors (*n* = 12)	SETD2 wt (*n* = 6)	SETD2 mt (*n* = 6)	*p* value (wt v mt)
Age (years)	71.67 ± 5.03	63.58 ± 7.76	61.00 ± 7.85	66.17 ± 7.41	
	*p* = 0.081		*p* = 0.378*		
Sex (males)	2 (66.67%)	9 (75%)	4 (66.67%)	5 (83.33%)	
	*p* = 0.661		*p* = 0.999		
SSIGN Score (4–8/ ≥ 9)		9/3	5/1	4/2	0.999
T-Stage (T1/T2/T3/T4)		4/0/8/0	2/0/4/0	2/0/4/0	1.000
Tumor Size (cm)		8.33 ± 4.08	9.20 ± 2.86	9.00 ± 4.88	0.933
Grade (2/3/4)		6/4/2	3/2/1	3/2/1	1.000
Necrosis (yes)		3 (25%)	1 (16.67%)	2 (33.33%)	0.999
Nodal Stage		N0	N0	N0	
M Stage (M1)		7 (58.33%)	3 (50%)	4 (66.67%)	0.999

^*^ Nonparametric test used

ITH was first examined at the pathologic level by evaluating H&E and IHC (H3K36me3 and 5mC) stains. Representative images are shown in Fig. 1A (bottom). A strong correlation between *SETD2* mutation and H3K36me3 levels has been established [28]; therefore, H3K36me3 is used as a surrogate for *SETD2* status given that SETD2 antibodies are not suitable for IHC. The oncoprint in Fig. 1B summarizes the extent of heterogeneity across various tumor characteristics. Of the five parameters we examined on an individual tumor region basis (grade, necrosis, SSIGN, H3K36me3, and 5mC), all displayed varying degrees of ITH within 9/12 of the tumors. For the remaining pathologic categories (varying on a per region basis), H3K36me3 levels were significantly underrepresented in *SETD2* mt tumors (*p* < 0.01), while SSIGN score (*p* = 0.174), tumor grade (*p* = 0.762), and necrosis (*p* = 0.325) did not differ between sequence confirmed *SETD2* wt and mt tumors. However, even for H3K36me3 IHC, tumors scored as *SETD2* mt by single region targeted sequencing contained multiple regions of H3K36me3 positivity (e.g., patient m12, Fig. 1B) and conversely, sequence confirmed *SETD2* wt tumors contained regions that were negative for H3K36me3 (e.g., patient w3, Fig. 1B). In fact, for two *SETD2* wt patients (w1 and w2), all regions stained for H3K36me3 were negative (Fig. 1B). 5mC IHC levels were also heterogeneous across 5/12 patient tumors and correlated positively with H3K36me3 level determined for each region by IHC. That is, H3K36me3-positive tumor regions were more likely to be positively stained for 5mC (*p* = 1.35 × 10^–9^, Fig. 1C). Additionally, SSIGN score exhibits associations with 5mC (*p* = 3.86 × 10^–3^), H3K36me3 (*p* = 3.65 × 10^–9^), and nuclear grade (*p* = 1.35 × 10^–9^). Tumor regions with low SSIGN score (0–7) are associated with less aggressive tumor traits such as 5mC and H3K36me3 positivity, as well as lower nuclear grade (Fig. 1C). Taken together these results reveal considerable heterogeneity within our 12 examined tumors at multiple pathologic levels. These results also reveal that while H3K36me3 IHC correlates well overall with *SETD2* mutation calls, there are limits to this association revealed by our regional analyses.

### Differential DNA methylation between normal and tumor samples reveals that inter-patient heterogeneity dominates the 5mC landscape

**Fig. 2:**
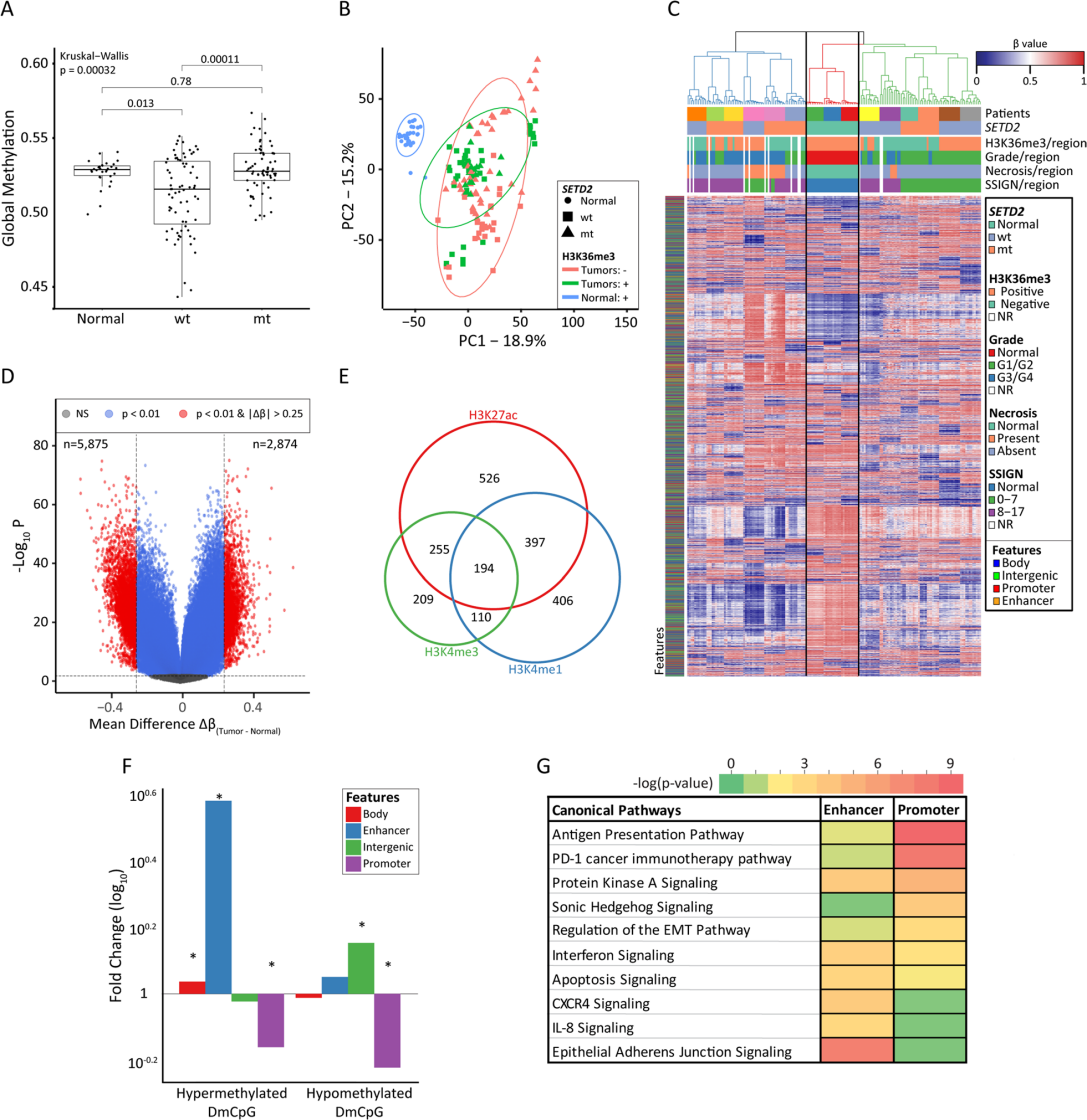
Defining differential DNA methylation among normal kidney and *SETD2* wt/mt ccRCC.** A** Global 5mC of normal kidney (*n* = 30) and ccRCC regions (*n* = 138, stratified by *SETD2* status). **B** PCA using all autosomal CpGs (*n* = 843,393). Each point represents a tissue region. Each region’s shape is representative of the *SETD2* status of the tumor it originates from, and the color is representative of the H3K36me3 IHC status of each region. **C** Unsupervised hierarchical clustering using the 5,000 most variable CpGs across the whole cohort. **D** Volcano plot of the differential analysis between tumor and normal samples. DMCpGs are represented by red points. **E** Venn diagram showing the number of DMCpGs (*n* = 2097) overlapping a selection of three histone marks: H3K27ac, H3K4me1, and H3K4me3. *N* = 6652 DMCpGs are not represented as they do not overlap with any of the three marks. **F** Bar plot showing the relative distribution of 2874 hypermethylated and 5875 hypomethylated DMCpGs, normalized to the distribution of CpGs in EPIC array, over four genomic features indicated. Enhancers are defined by loci overlapping with H3K27ac and H3K4me1, but not H3K4me3. CpGs mapped to TSS1500, TSS200, and 5’ UTR, in the EPIC manifest, are considered promoter CpGs. The asterisk indicates a significant distribution difference of the respective feature and the EPIC array total**.** Y-axis—relative difference (log_10_) of each feature. **G** Ontology of enriched pathways derived from 579 and 926 genes linked to the 397 active enhancer and 768 active promoter (defined as positive for H3Kme3 based on normal kidney) DMCpGs, respectively, from panel F, using IPA. The color bar represents the *p* values as −log_10_

5mC was measured using the Illumina EPIC (850 k) array from 30 samples derived from 3 normal kidneys (normal (N), no cancer, n = 10 regions/kidney) and 138 samples originating from multi-region sampling of 12 ccRCCs (~ 12 regions/tumor (T)). A comparison of global methylation between the normal and tumor groups stratified by *SETD2* mutation status shows significant differences among most groups (Fig. 2A). Specifically, the *SETD2* wt group is globally hypomethylated relative to normal kidney and the *SETD2* mt groups; surprisingly, overall 5mC of the *SETD2* mt group is not significantly different from normal kidney samples. Additionally, we examined methylation stratified by genomic feature, which revealed that gene bodies, promoters, intergenic regions, and enhancers (defined as regions with overlapping H3K27ac and H3K4me1 peaks, but lacking H3K4me3 in normal kidney), are all differentially methylated between the three groups, highlighting that global differential methylation is not driven by any specific feature (Additional file 2: Fig. S1). Principal component analysis (PCA) using all CpGs on autosomal chromosomes shows a segregation between normal and tumor regions, and a tendency for H3K36me3-negative tumor regions to segregate from the center of the plot and appear overall more variable (larger spread, red squares and triangles, Fig. 2B). Furthermore, unsupervised hierarchical clustering employing the top 5,000 most variable CpGs segregated normal and tumor regions entirely on a per patient basis (Fig. 2C), indicating that 5mC differences between patient tumors are greater than within individual patient tumors (patients are indicated by the top-most color bar in Fig. 2C).

We performed methylation differential analysis between normal kidney and ccRCC. We observe 166,411 differentially methylated CpGs (DMCpGs) using a Δβ cutoff of ± 0.1 and a *p* value < 0.01 (Additional file 2: Fig. S2). Genes ranking as highly differentially methylated include *PRDM16* (*n* = 152 CpGs) and *DPP6* (*n* = 72 CpGs) which, when hypermethylated, are associated with poor outcome in ccRCC [15, 29]. These loci in our tumor samples show extensive promoter hypermethylation, consistent with an inverse correlation of promoter 5mC and gene expression for these loci in TCGA-KIRC (Additional file 2: Fig. S3A and S4A, tracks 3–4). To investigate DMCpGs more stringently, we first raised the Δβ to ± 0.25 (*p* value < 0.01), yielding 8749 DMCpGs between normal and tumor samples (Fig. 2D). Of the 8749 DMCpGs shown in Fig. 2D, 2874 CpGs are hypermethylated and 5875 CpGs are hypomethylated (their distribution by feature is summarized in Additional file 1: Table S4). Using ChIP-seq data from the Epigenome Roadmap, we defined a set of normal kidney active enhancers as regions intersecting with active regulatory region histone marks H3K27ac and H3K4me1, but not the active promoter-associated H3K4me3 mark. Using these regions, we identified 397 DMCpGs between normal and tumor linked to enhancers (Fig. 2E). The 8749 DMCpGs were also differentially enriched among promoter, body, intergenic, and enhancer features, with promoter changes underrepresented and enhancer DMCpGs over-represented, relative to all CpGs on the EPIC array (Fig. 2F), indicating that normal kidney enhancers are targets for epigenome deregulation in ccRCC, particularly for hypermethylation events. Ontology analysis reveals that ccRCC DMCpGs map to genes linked to tumor driver and anticancer therapy pathways including PD-1 immunotherapy, invasion/metastasis (epithelial-mesenchymal transition (EMT) and CXCR4 signaling), and inflammation (interferon and IL8 signaling, Fig. 2G).

### The normal kidney displays low levels of regional epigenetic heterogeneity

**Fig. 3:**
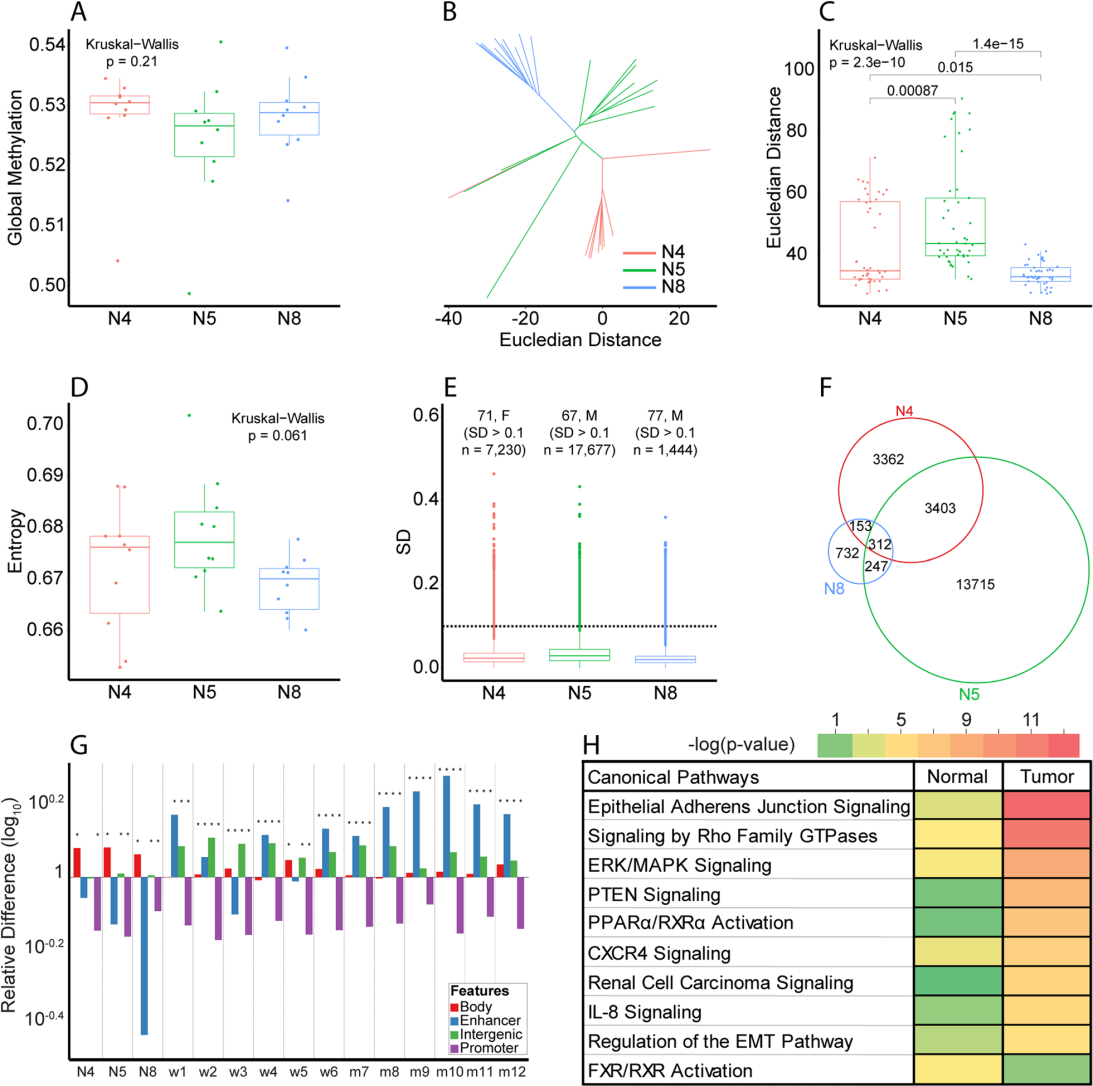
Quantifying and localizing epigenetic heterogeneity within normal kidney tissue.** A** Boxplot comparing global 5mC between three normal kidneys, 10 regions each. Each dot represents the mean methylation of 843,393 CpGs per region. **B** Phyloepigenetic tree showing the branching of 30 normal regions obtained from 3 normal kidneys. **C** Boxplot comparing the Euclidian distances within each normal kidney. Each dot represents the distance between any two samples originating from the same normal kidney. **D** Boxplot comparing entropy among the same normal kidney regions. Each dot represents the entropy of a sample calculated for 843,393 CpGs. **E** Boxplot showing the SD of each CpG within each normal kidney. Age and sex of the donors are indicated along with the number of highly variable (SD > 0.1) CpGs in each kidney. The dotted red line intersects the y axis at *y* = 0.1 and establishes our threshold between high and low variability. **F** Venn diagram showing the overlap of the highly variable CpG group between the three normal kidneys. **G** Bar plot showing the relative feature distribution of the highly variable CpGs of each normal kidney (*n* = 3) and the ccRCCs (*n* = 12) when normalized to the distribution of all CpGs on the EPIC array over four genomic features indicated. Enhancers and promoters are defined as in Fig. 2. The asterisk indicates a significant distribution difference of the respective feature and the EPIC array total**.** Y-axis—relative difference (log_10_) of each feature. **H** Ontology of enriched pathways derived from 574 and 2027 genes linked to the 368 active enhancers in the normal group and the 5,000 most variable active enhancer CpGs from the tumor group, respectively, from panel G, using IPA. The color bar represents the p-values as −log_10_

We next examined heterogeneity in normal kidney, which could be subject to age or environmental factors, by sampling ten geographically distinct regions from three normal kidneys (Additional file 1: Table S5) to assess global 5mC, entropy, and Euclidian distance. This analysis shows there are no significant global methylation differences among the normal kidney regions or across the three donors (Fig. 3A). This result also excludes the possibility of selection bias during sample selection and constitutes a foundation on which to draw a normal baseline. Upon stratification of CpGs into their respective features, only enhancer CpGs were different among the normal kidneys (Additional file 2: Fig. S5), which may be reflective of differing environmental exposures since the age of the donors did not differ significantly. Euclidian geometry measures the distance between organisms based on triangle inequality. It can be used to assess genome-wide divergence between samples, where a large Euclidian distance is reflective of greater difference between samples [30]. In a phyloepigenetic tree, all regions of an individual donor kidney cluster together (or branch from a common point). More specifically, kidney regions from NDK8 tend to be closest to each other with the shortest intra-tissue Euclidian distances, followed by NDK4, and NDK5 whose regions are the most spread (Figs. 3B, C).

To further examine heterogeneity, we measured entropy for all 10 regions from each normal kidney using a modified Shannon entropy for 5mC data [31]. We observed no significant entropy differences globally among the three normals (Fig. 3D), but did find, when stratified by feature, that gene body and promoter CpGs showed significantly different entropies (Additional file 2: Fig. S6). We therefore queried intra-kidney variability by measuring the standard deviation (SD) of each CpG and observed overall very low 5mC variability (median SD of CpGs being lower than 0.1, Fig. 3E). The number of highly variable CpGs (SD > 0.1) varied between the three kidneys with NDK5 having the largest number (*n* = 17,677 CpGs; 2.10%) and NDK8 having the smallest number (*n* = 1444 CpGs; 0.17%). This observation is concordant with results in Figs. 3B, C, where NDK5 and NDK8 have the longest and shortest intra-tissue Euclidian distances, respectively. Additionally, the highly variable CpGs tend to be unique to their respective patient with only 41.16% shared with at least one other normal kidney (Fig. 3F), suggesting a distinct randomness to this set of normal kidney variable CpGs. Variable CpGs are enriched in gene bodies but depleted for enhancers and promoters (Fig. 3G). Interestingly, when examining the highly variable CpGs in the 12 ccRCCs (with SD > 0.1), this feature-related trend completely reverses, with an enrichment of the most variable tumor CpGs in enhancers and reduced gene body enrichment (Fig. 3G). Finally, we examined 574 genes associated with highly variable enhancer CpGs in normal kidney tissue, and 2027 genes associated with the 5000 most variable enhancer CpGs in tumors. Ontology revealed that enhancer CpGs from the normal group are weakly associated with pathways commonly linked tumorigenesis (e.g., EMT and renal carcinoma signaling), unlike the tumor enhancer CpGs that were enriched for cancer-associated pathways like MAPK, PTEN, and CXCR4 signaling (Fig. 3H) [32, 33]. Taken together, these findings indicate an overall low level of intra-kidney methylation variability, and that the modest number of CpGs that do display heterogeneity are largely unique to each patient.

### Epigenetic heterogeneity is markedly elevated in tumors and impacted by *SETD2* status

**Fig. 4:**
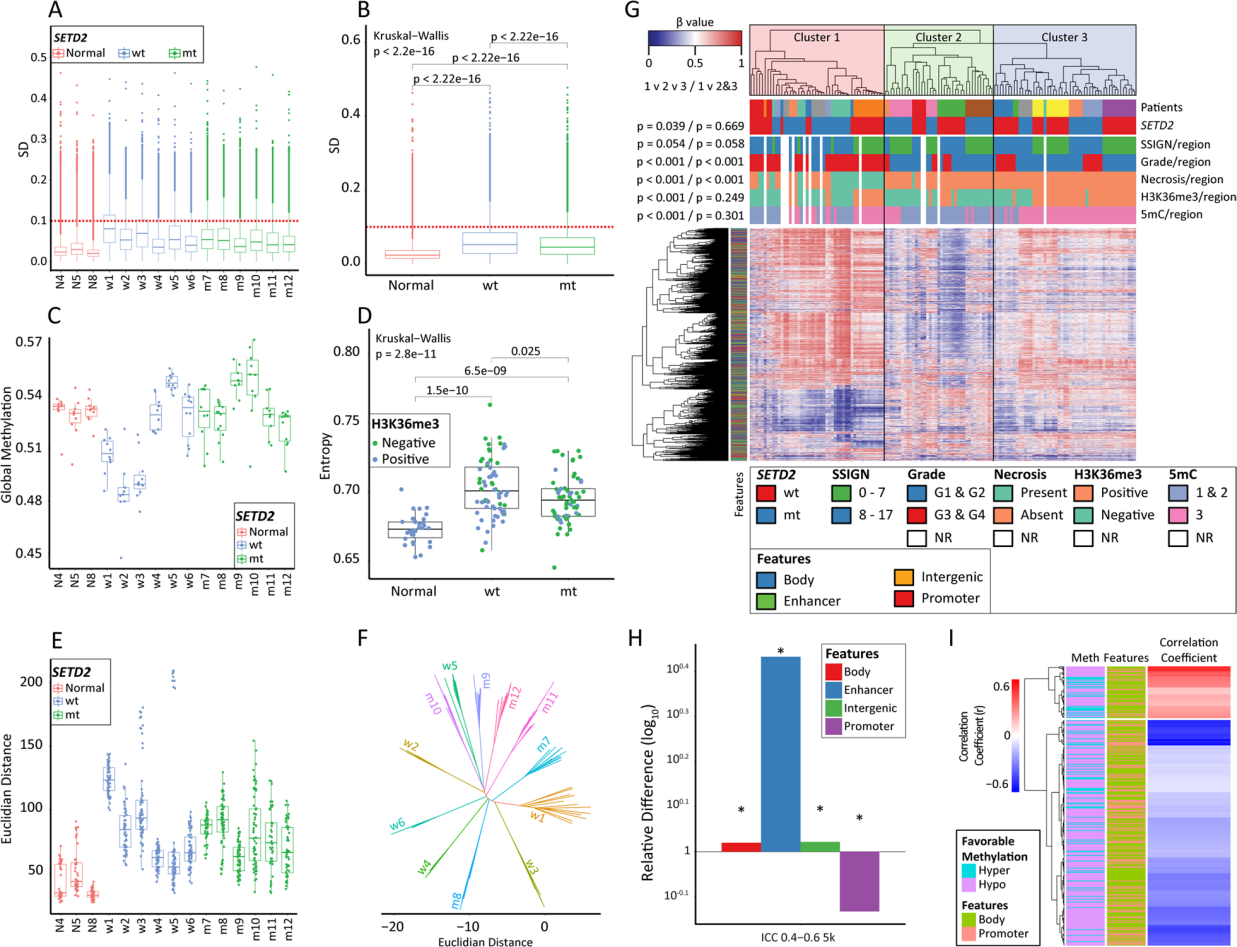
Defining epigenetic heterogeneity within cRCC.** A** Boxplot of the SD of each CpG within sampled regions from each patient. The red dashed line intersects the y axis at y = 0.1. CpGs with a SD greater than 0.1 considered heterogeneous. **B** Boxplot of the SD of each CpG within all regions of normal kidney and ccRCCs stratified by their *SETD2* status. The red dashed line intersects the y axis at y = 0.1. CpGs with a SD greater than 0.1 considered heterogeneous. **C** Global 5mC of sampled regions from each patient. Each dot represents the mean methylation of a sample using 843,393 autosomal CpGs. **D** Entropy comparison of normal kidney and *SETD2* wt/mt tumor regions. Dots represent the entropy calculated using the amended Shannon entropy for 843,393 CpGs. Dot colors reflect the H3K36me3 status of each tumor/normal region. **E** Euclidian distance comparison of sampled regions from each patient using 843,393 autosomal CpGs. Dots represent the distance between any two samples originating from the same patient. **F** Phyloepigenetic tree of the 5000 most variable CpGs among tumor samples. **G** Supervised hierarchical clustering of the 5,000 most variable CpGs within the ICC 0.4–0.6 group forms three major clusters. P-values result from the *χ*^2^ analysis of all clusters against each other and the comparison of cluster 1 against the combination of clusters 2 and 3. CpG features are indicated by the colored bar to the left of the heatmap. **H** Relative feature distribution of the 5,000 most variable CpGs within the ICC 0.4–0.6 group when normalized to the distribution of all CpGs on the EPIC array over four genomic features. The asterisk indicates a significant distribution difference of the respective feature and the EPIC array. Y-axis – relative difference (log_10_) of each feature. **I** Heatmap showing the correlation coefficients (r) of 392 KIRC survival-linked CpGs significantly associated with the expression of their respective genes. The dendrogram separates the CpGs with positive (*n* = 73; red) from the CpGs with negative (*n* = 319; blue) correlations. The side bars correspond to the methylation status associated with better survival, and the feature of the CpG. There is a significant over representation (*p* = 0.011) of CpGs associated with better survival when hypomethylated among the CpGs inversely correlated with the expression of their respective genes. There is no feature distribution difference between the two CpG clusters

To assess intratumor heterogeneity, we applied the same measures used for the normal kidney regions, to the 138 distinct tumor regions obtained from 12 ccRCC patients. Tumor regions possess significantly higher variability than normal kidney (Figs. 4A, B). This difference in variability is also observed when tumors are stratified according to *SETD2* genetic status (Fig. 4B), with the median SD of CpGs in the *SETD2* wt ccRCC group being significantly higher than that in the *SETD2* mt group of tumors (*p* < 2.2 × 10^–16^). The standard deviation of intratumor global 5mC is significantly greater than the standard deviation of intra-tissue normal kidney 5mC (µSD_Tumor_ = 0.015, µSD_Normal_ = 0.009, *p* = 0.016). This difference, however, disappears when stratified by *SETD2* status (µSD_wt_ = 0.016, µSD_mt_ = 0.013, *p* = 0.291, Fig. 4C).

As shown in Fig. 4D, tumors have significantly higher entropy than normal kidney, indicating an elevated level of disorder in ccRCC. Furthermore, we observe a wider spread in entropy values for the tumor group as a whole, reflecting more diverse methylomes present within the same tumor (presumably corresponding to a more phenotypically diverse tumor cell population). Higher grade and presence of metastasis are also associated with higher entropy (Additional file 2: Fig. S7). Analysis of entropy associations with other clinico-pathological characteristics (age, tumor size, necrosis, etc.) can be found in Additional file 2: Fig. S7. Moreover, *SETD2* wt tumors have a significantly higher entropy (p = 0.025) than *SETD2* mt tumors (Fig. 4D), but when stratified by H3K36me3 IHC status, no significant difference is observed (*p* = 0.220). We then examined the Euclidian geometry within all patients and observed that tumors have significantly higher medians and wider ranges of Euclidian distances than those observed among the normal kidney regions (Fig. 4E). These results reveal greater divergence within tumor samples than normal kidney, and within tumors *SETD2* wt ccRCC manifests with significantly larger Euclidian distances than *SETD2* mt ccRCC (µED_wt_ = 86.88, µED_mt_ = 77.51, *p* = 1.98 × 10^–5^).

### Identification of a distinct group of CpGs linked to intratumor heterogeneity

We next sought to identify CpGs driving heterogeneity across our cohort of 12 tumors. Using the 5,000 most variable CpGs in the ccRCC group of samples/regions, it was not possible to identify heterogeneity drivers since tumor regions segregated purely by patient (Fig. 4F). This finding indicates that this traditional method of analysis fails to filter out inter-patient differences, which dominate the 5mC landscape. To identify CpGs with high variability driven by ccRCC as opposed to patient genetics and/or environmental exposures, we calculated the intraclass correlation coefficient (ICC) for all autosomal CpGs on the EPIC array for this sample set [34, 35]. CpGs with high ICC show high inter-individual variation (differences are greater between two samples obtained from two individuals, compared to two samples obtained from the same individual: genetically driven, for example). On the other hand, CpGs with low ICC reflect high intraclass variation and low inter-individual variation (differences are greater between two samples obtained from the same individual than two samples obtained from different individuals: intratumor epigenomic variation irrespective of patient genetics/environment). A depiction of the inter- and intra-patient variability for representative CpGs based on different ICC levels is shown in Additional file 2: Fig. S8. We distributed CpGs into three groups based on their ICCs (ICC < 0.4, *n* = 461,927 CpGs, ICC 0.4–0.6, *n* = 267,991 CpGs, and ICC > 0.6, *n* = 113,475 CpGs). The behavior and properties of CpGs in each of the three ICC groups are significantly different. For example, CpGs within the ICC 0.4–0.6 group are significantly hypermethylated, while CpGs within the ICC > 0.6 group are significantly hypomethylated (Additional file 2: Fig. S9A). This differential methylation is also observed within each of the ICC groups when tumors are stratified by *SETD2* mutational status or genomic feature (Additional file 2: Figs. S9B-C). In terms of genes/biological processes, enhancer-associated CpGs in the low ICC group are enriched for xenobiotic metabolism and inhibition of matrix metalloprotease pathways, while the enhancers in the high ICC group are enriched for sonic hedgehog and interferon signaling (not shown). For the intermediate ICC group, we compared CpGs in enhancers relative to promoters (determined by H3K4me3 presence), which revealed that enhancer CpGs are enriched for protein kinase A and PPAR signaling pathways while promoters are enriched for p53 signaling and antigen presentation pathways (Additional file 2: Fig. S9D). To drill down on CpGs of interest, we performed unsupervised hierarchical clustering using the 5,000 most variable CpGs from each of the three ICC CpG sets (ICC < 0.4, Additional file 2: Fig. S10A; ICC 0.4–0.6, Fig. 4G; and ICC > 0.6, Additional file 2: Fig. S10B). The ICC < 0.4 set yields no distinct clusters and there is a high degree of intermixing of tumor regions across all patients (Additional file 2: Fig. S10A). In contrast, CpGs from the ICC > 0.6 set cluster all tumor regions based on their patient of origin, thus masking intratumor diversity which drives ITH (Additional file 2: Fig. S10B). Unlike the other two sets, the ICC 0.4–0.6 group CpGs segregate tumor regions into three distinct groups, and tumor region clustering is not dominated by patient of origin, indicating that this intermediate ICC range does indeed discover CpGs with high inherent epigenetic ITH (Fig. 4G). Cluster 1, dominated by hypermethylation events, is overrepresented for tumor necrosis and higher nuclear grade (G3 and G4) regions. Cluster 2 is enriched for hypomethylation events, while cluster 3 is intermediate. The 5,000 most variable CpGs from the ICC 0.4–0.6 group are also enriched for normal kidney enhancers and depleted of promoters (Fig. 4H, Additional file 1: Table S6). In summary, CpGs with an intermediate level of ICC represent markers of ITH with ccRCC population-wide relevance. The different ICC group CpGs also present with distinct properties, genomic localizations, and links to clinical phenotypes.

To examine properties of the 5000 most variable CpGs in the ICC 0.4–0.6 list in a larger independent set of samples, we examined their 5mC, expression, and survival relationships using TCGA’s KIRC database, as diagrammed in Additional file 2: Fig. S11. After overlapping the two datasets, 1945 out of 5000 CpGs are shared with the Illumina 450 k array (used for the KIRC methylation analysis) and 819 out of 1945 CpGs are significantly associated with better ccRCC-specific survival (Additional file 1: Table S7). In addition, the methylation of 392/819 CpGs is significantly correlated with expression of their respective gene(s) (Fig. 4I). We also observe a significant (*p* = 0.011) overrepresentation of CpGs associated with better survival when hypomethylated, among the CpGs negatively correlated with expression of their respective genes. Collectively, 66% (*n* = 172) of these CpGs are in the body of the gene, and 34% (*n* = 89) are in the promoter. Genes linked to CpGs within the ICC 0.4–0.6 group include *PRDM16* and *DPP6*, which were identified earlier to be among the most hypermethylated in our ccRCC cohort (Additional file 2: Fig. S3B and S4B). The expression of *PRDM16* and *DPP6* is downregulated in TCGA-KIRC, and the promoters of these genes are significantly hypermethylated (with both parameters linked to poorer RCC-specific survival). Higher nuclear grade and necrosis are also linked to *PRDM16* and *DPP6* promoter hypermethylation (Additional file 2: Figs. S3B-G and S4B-G). Taken together, our data suggest that the *PRDM16* and *DPP6* genes play an important role in ccRCC onset and progression, consistent with work from others [36, 37].

### Phyloepigenetic analysis of enhancers reveals novel putative drivers of ccRCC

**Fig. 5:**
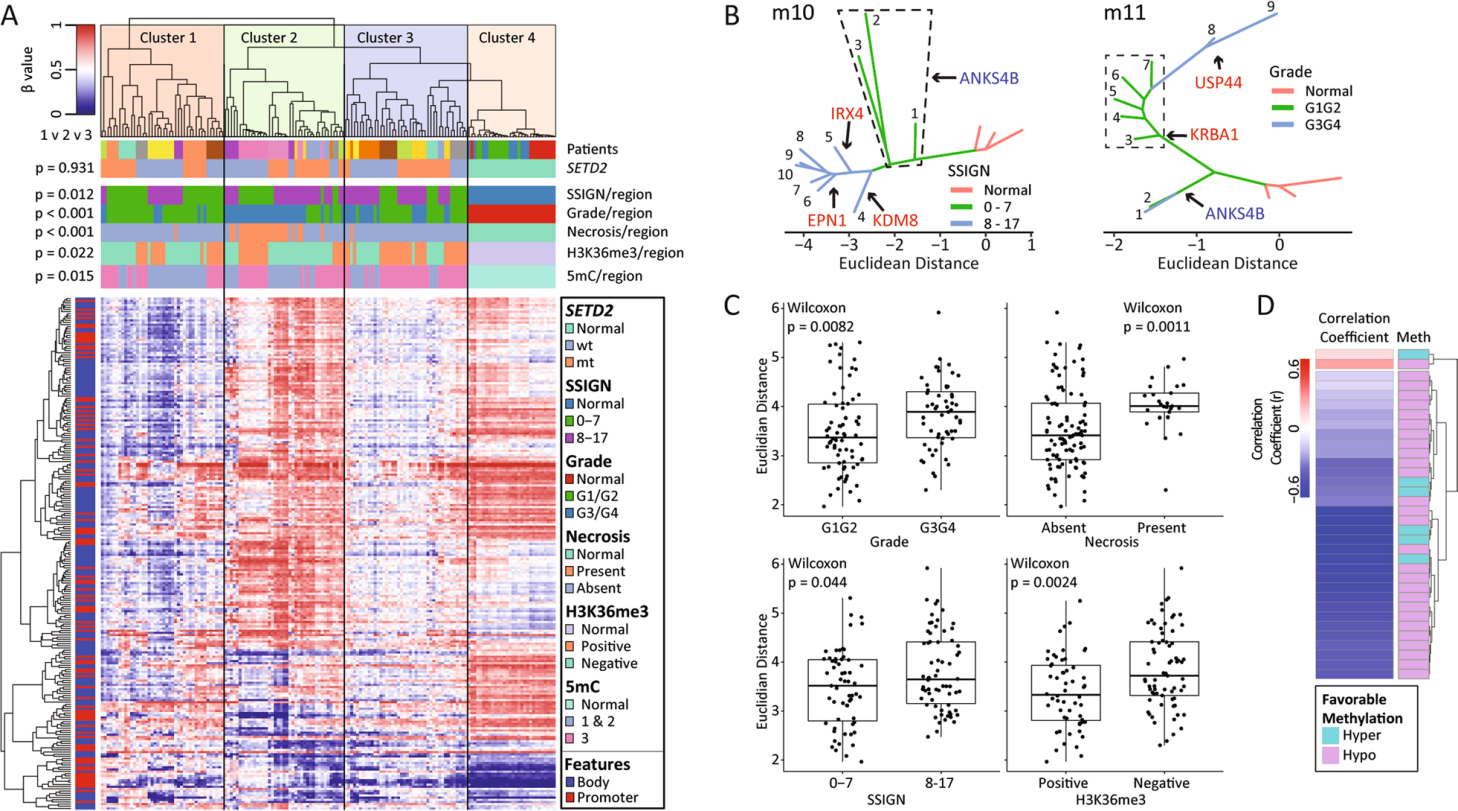
Enhancer-associated CpGs as drivers of ITH in ccRCC. **A** Heatmap resulting from supervised hierarchical clustering of 236 enhancer-associated CpGs identified using GeneHancer that are predicted to influence expression of their respective gene. Four major clusters are formed. The p-values are the result of χ^2^ analyses of clusters 1, 2, and 3 against each other. The color bar at the left indicates the genomic feature of each CpG. **B** Two representative phyloepigenetic trees showing evolutionary methylation changes of regions from the same tumor, with progression from the normal baseline into low grade and then high-grade regions. Major evolutional intervals are highlighted and identify genes with the highest number of CpG methylation changes (red = hypermethylation; blue = hypomethylation). **C** Boxplots comparing the average Euclidian distance between normal kidney and ccRCC regions with various clinico-pathological parameters (grade, necrosis, SSIGN, and H3K36me3). Each point represents the average distance between a tumor region and the normal kidney samples. **D** Heatmap showing the correlation coefficients (*r*) of 34 KIRC survival-associated CpGs significantly associated with expression of their respective genes. The dendrogram separates CpGs with positive *r* (*n* = 2; red) from CpGs with negative r (*n* = 32; blue) correlations. The side bar indicates whether the methylation status is linked to more favorable survival outcome

To identify common targets of epigenetic deregulation that may drive ITH and impact ccRCC biology, we conducted singular value decomposition (SVD) analysis on CpGs linked to enhancers using the GeneHancer database of pan-tissue enhancers and their predicted gene targets (*n* = 435,104 CpGs) [38]. For each tumor, SVD ranks CpGs in order of their contribution to the sample deconvolution. We selected the top 5,000 CpGs from each tumor per SVD ranking and determined the frequency of highly ranked CpGs across our panel of tumors, which yielded 435 enhancer-associated CpGs common to any four or more tumors. Of these, 236 CpGs are located in the body or promoter of the gene they are predicted to regulate (Additional file 2: Fig. S12A; Additional file 1: Tables S8–9). Applying this subset of CpGs to our entire cohort in hierarchical clustering resulted in four distinct clusters that segregated all normal (cluster 4) from all tumor regions and further divided the ccRCC regions into three groups; cluster 1 = overall hypomethylated; cluster 2 = hypermethylated; cluster 3 = intermediate methylation level (Fig. 5A). Among the ccRCC clusters, hypermethylated cluster 2 showed an overrepresentation of regions with necrosis and high nuclear grade (G3/G4), while hypomethylated cluster 1 was overrepresented for low 5mC (1/2) and low SSIGN score (0–7). H3K36me3-positive regions were enriched in the intermediate methylation cluster 3.

The 236 enhancer-associated CpGs were able to discriminate among tumor regions and across patients; therefore, we examined how they cluster regions of the same tumor within an individual patient. Using an evolutionary differential analysis between phyloepigenetic-based tree branches of the same tumor, we identified genes with a unique methylation signature that contribute to each tree’s branching pattern by treating normal samples as baseline and identifying differentially methylated (Δβ > 0.1) CpGs between every group of branches and all branches preceding them. This allowed for discovery of gradual and evolutionary methylation changes in each tumor for the 236 enhancer CpGs (Fig. 5B, Additional file 2: Fig. S13). For example, *USP44*, a ubiquitin-specific protease that is hypermethylated in the most terminal/progressed branches of patient m11 (Fig. 5B, left), is known to be downregulated and hypermethylated in ccRCC, and *USP44* expression is negatively correlated with ccRCC stage, grade, and patient survival [39]. Consistent with this, higher expression of 5/13 genes shown across phyloepigenetic trees in Additional file 2: Fig. S13, including *USP44,* is associated with more favorable survival in TCGA-KIRC (Additional file 2: Fig. S14). On average, tumor regions with more aggressive characteristics (i.e., high grade/SSIGN, necrosis) show the greatest Euclidian distance from normal kidney in their respective trees (Fig. 5C). To further understand links between the ITH-driven enhancer CpGs and primary ccRCC, we validated their 5mC/expression survival associations using TCGA-KIRC data (method summarized in Additional file 2: Fig. S12B). Of the 236 CpGs from the EPIC array, 77 are shared with the Illumina 450 k array (used for TCGA-KIRC 5mC analysis), and of these, 49 out of 77 CpGs are significantly associated with better ccRCC survival (Additional file 1: Table S10). In addition, expression of the genes linked to 34 of these 49 CpGs is significantly correlated with their methylation status (28 genes in total, including *ANKS4B*, *KRBA1*, and *USP44*) (Fig. 5D). Taken together, the 236 heterogeneity-driven enhancer-linked CpGs have the power to segregate individual tumor regions by clinical properties and show that more aggressive regions tend to be most distinct, or progressed evolutionarily, from less aggressive ccRCC regions and normal kidney.

### Intratumor epigenetic heterogeneity and copy number variation (CNV)

**Fig. 6:**
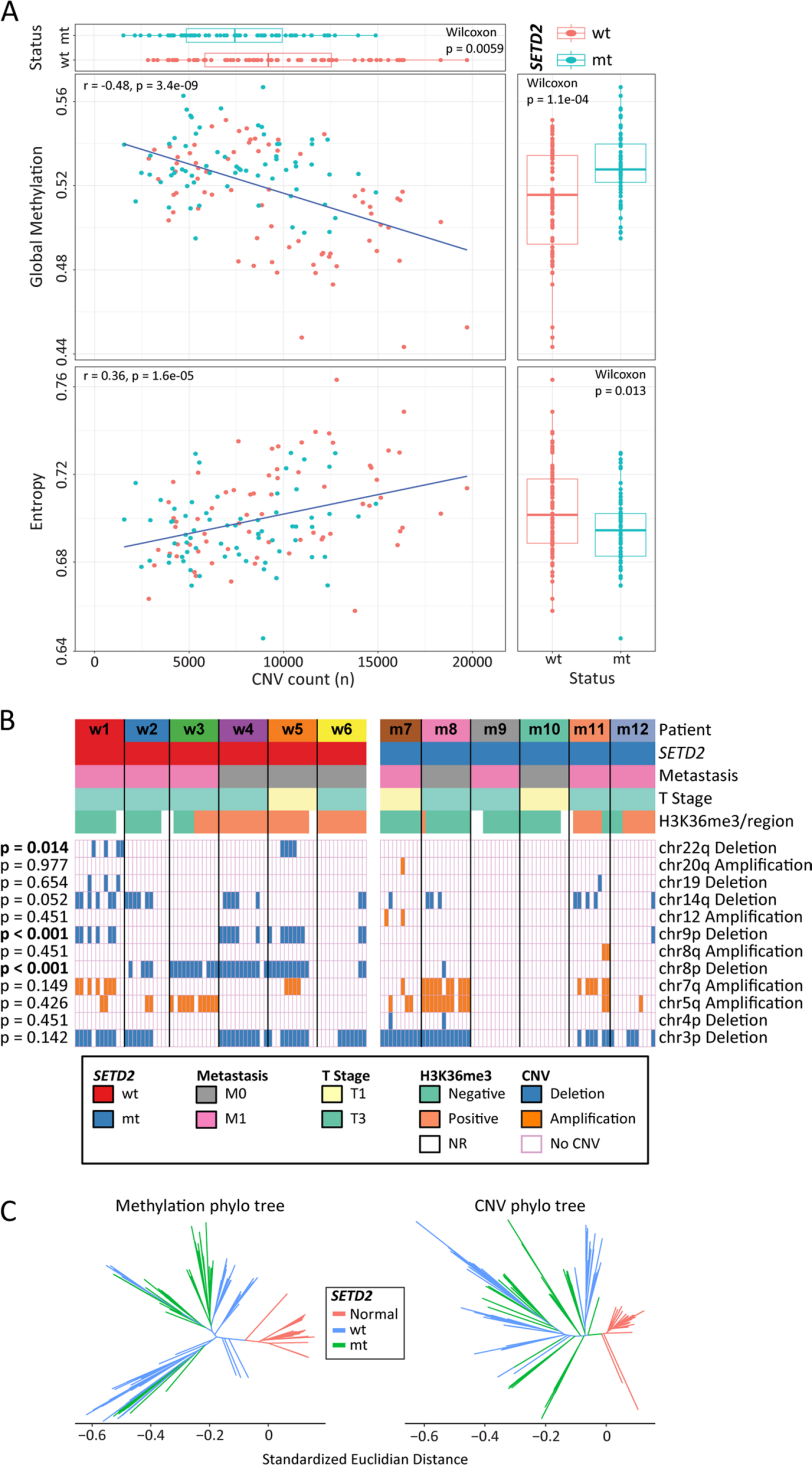
Interplay between copy number level ITH, 5mC, and clinical parameters. **A** Scatterplots showing the correlation between CNV counts and global 5mC, and CNV counts and entropy, for ccRCC regions. Each dot represents a tumor region and is colored according to the *SETD2* status of the tumor of origin. The barplot at the top of the panel shows that regions of *SETD2* mt tumors have a significantly smaller number of CNVs. The side panels show a significant global hypermethylation in *SETD2* mt tumors and a significantly higher entropy in *SETD2* wt tumors. **B** Oncoprint showing presence and absence of CNVs associated with ccRCC as per Gerlinger et al. [1] and Gulati et al. 44] in our cohort of 12 tumors. Gross tumor characteristics (*SETD2* mutational status, metastasis, and T stage) of all 12 tumors, and a region-specific result (H3K36me3 IHC status) are also shown. Tumors are grouped according to their *SETD2* mutational status (wt to the left and mt to the right). *P* values are the outcome of χ^2^ comparison of each CNV between *SETD2* wt and mt tumors. Some tumor regions could not be scored (‘NR’) and are left white. **C** Phylo(epi)genetic trees corresponding to the 5mC and CNV datasets. The 5mC tree is drawn using the standardized Euclidian distance measured for the methylation of 843,393 CpGs, and the CNV tree is drawn using the standardized Euclidian distance measured for the log2 signal intensities for 25,752 loci

Given that copy number variation (CNV) is associated with poor outcome in multiple cancer types [40] and can be derived from EPIC array data [41], we explored ITH at the CNV level and its interplay with epigenetic ITH. We observe variation in copy number within individual tumors overall and a significant difference in the number of CNVs between normal kidney, *SETD2* wt, and *SETD2* mt ccRCC regions (*p* < 2.2 × 10–16, Additional file 2: Fig. S15A). A significant difference in CNV is also observed between *SETD2* wt and *SETD2* mt tumor regions (*p* = 1.4 × 10^–9^, Fig. 6A, top panel), with higher CNV in the former. Overall, there was a negative correlation between global methylation and the number of CNV events (*r* = − 0.48, *p* = 3.4 × 10^–9^; Fig. 6A, top panel), consistent with previous findings in other tumor types [42, 43]. In contrast to global methylation, the number of total CNVs (gains and losses) positively correlates with 5mC entropy (*r* = 0.36, *p* = 1.6 × 10^–5^; Fig. 6A, bottom panel); reinforcing the notion that the level of chaos (entropy) in the methylome increases with the frequency of genetic changes.

CNV has been examined previously in ccRCC with several amplifications and deletions linked to clinical outcome, including chromosome 9p deletion [1, 44]. We focused on 12 of these ccRCC CNV hotspots and quantified their amplification or deletion status at the level of the chromosome. A detailed listing of all CNVs for each tumor region is provided in Additional file 1: Table S11. Our results indeed show ITH for this group of clinically relevant CNVs, with a significant overrepresentation of chr8p deletion (*p* < 0.001), chr9p deletion (*p* < 0.001), and chr22q deletion (*p* = 0.014) among regions corresponding to *SETD2* wt tumors (Fig. 6B), suggesting that the presence of *SETD2* mutation/loss of H3K36me3 influences genomic stability or drives tumor development down a more epigenetically driven pathway less reliant on genome instability.

Having established ITH at the 5mC and copy number levels within the same tumor regions, we sought to understand if one of these parameters was more dominant in terms of ITH and the subsequent branching structure of phylo(epi)genetic trees. Since the scales of CNV and methylation data are different, we standardized the Euclidian distances of each dataset separately before conducting a comparison by using the method described in Hua et al. (2020) [45]. This analysis shows that the phyloepigenetic tree based on 5mC is significantly larger than the phylogenetic tree based on CNV, with a median standardized Euclidian distance fold difference of 1.41 (*p* < 0.001; Fig. 6C). This reflects a more diverse environment in the methylome than the genome, at least at the level of CNV. Furthermore, the mean Euclidian distance between any tumor region and normal kidney region is significantly larger in the methylation tree compared to the CNV tree (Additional file 2: Fig. S15B). Lastly, by plotting the standardized Euclidian distances between ccRCC and normal kidney regions for the two trees stratified by clinical and pathologic variables (Additional file 2: Figs. S15C–F), we observe that tumor regions associated with more aggressive phenotypes (high grade, necrosis, and high SSIGN score) have on average significantly longer standardized Euclidian distances from normal samples in the 5mC tree, while displaying no difference in the CNV-based tree. Taken together, these findings reveal that CNV shows antithetical relationships with methylation and entropy, and that overall, the methylome is a greater source of variability than CNV within ccRCC.

### Translational applications of epigenetic ITH to understand ccRCC biology

**Fig. 7:**
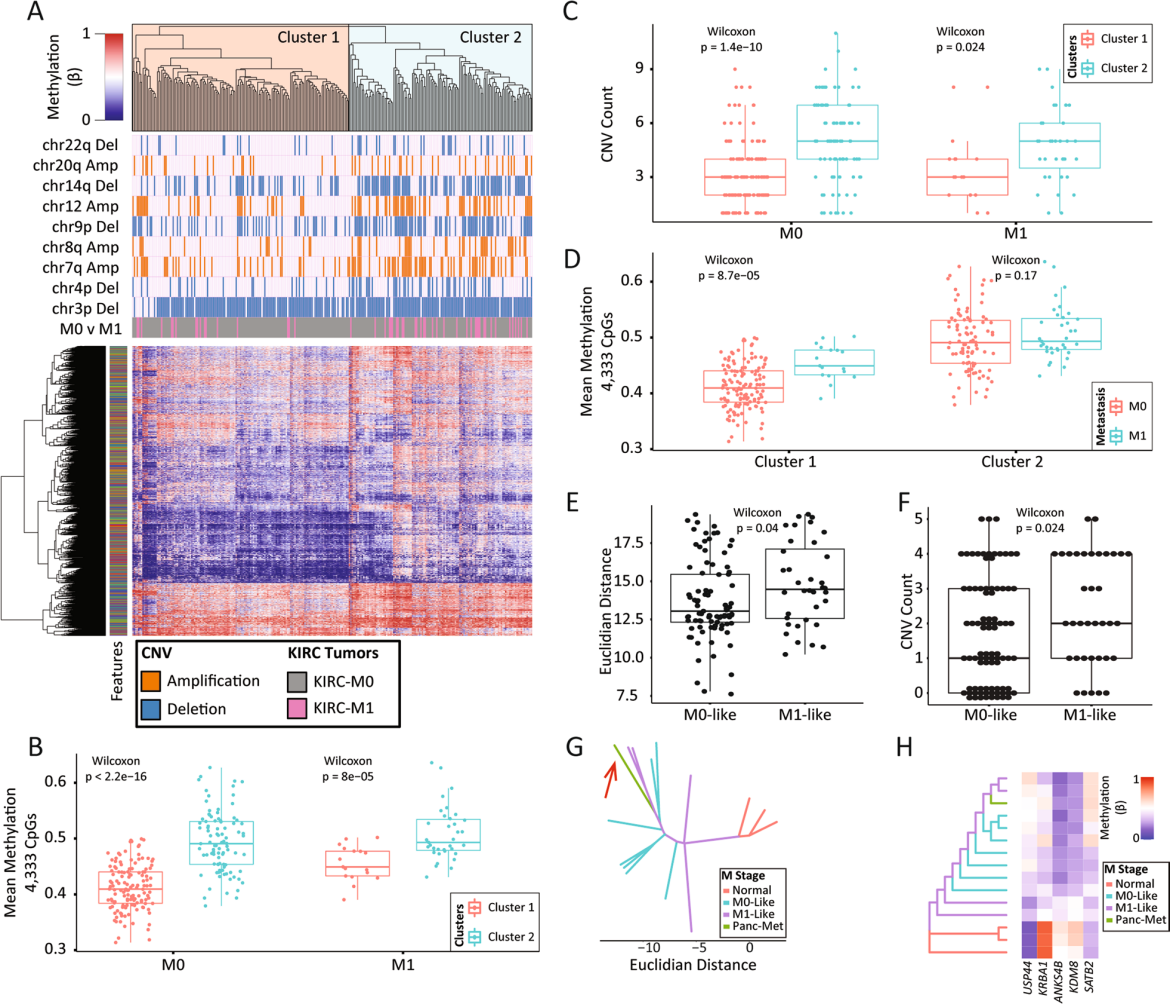
Translational application of epigenetic ITH to understand ccRCC metastasis. **A** Unsupervised hierarchical clustering of TCGA’s KIRC cohort using the 4333/5000 most variable CpGs that overlap with the EPIC array and do not have any missing data across samples. **B** Comparison of mean 5mC of the 4333 CpGs of the M0 tumors in cluster 1 to those in cluster 2, and the M1 tumors in cluster 1 to those in cluster 2. **C** Boxplot showing the over-representation of CNVs in cluster 2 irrespective of the metastasis status. Dots represent TCGA-KIRC samples, and the y-axis indicates the number of CNVs from a published set of CNVs linked to clinical features [1, 44]. **D** Comparison of mean 5mC of the 4333 CpGs of the M0 to the M1 tumors in cluster 1, and then the M0 to the M1 tumors in cluster 2. **E** Euclidian distance comparison between M0-like samples in our cohort and the normal kidneys, and the distance between the M1-like samples and the normal kidneys. **F** Difference in the number of observed CNVs in our M0-like and M1-like samples. **G** Phyloepigenetic tree for tumor w3 drawn using the 4333. M0-like and M1-like branches are colored differently. A metastatic sample obtained from the pancreas is included in this tree (red arrow) and branches off with two M1-like regions most distant from the normal kidney. **H** Phyloepigenetic tree for tumor w3 drawn using 236 enhancer-associated CpGs derived from Fig. 5. M0-like and M1-like branches are colored differently. The heatmap represents the average 5mC of the enhancer CpGs mapped to genes indicated on the tree in Additional file 2: Fig. S13: *USP44* (*n *= 3), *KRBA1* (*n* = 4), *AKNS4B* (*n* = 5), *KDM8* (*n* = 4), and *SATB2* (*n* = 2)

While ITH impacts treatment response, the major cause of ccRCC mortality results from metastasis. Given this fact, and that few genetic drivers of metastasis have been identified [22], we examined our epigenetic ITH data to probe for links between regional 5mC variation and ccRCC metastatic potential. We began by querying TCGA-KIRC data by unsupervised hierarchical clustering and found that using methylation at the 5000 most variable CpGs segregated primary ccRCC that metastasized (M1) from primary ccRCC that did not (M0, *p* = 5.23 × 10^–4^; Additional file 2: Fig. S16). Given the imperfect overlap between the 450 K (used by TCGA) and EPIC arrays (this study), integration of this result with our methylation data resulted in 4333 out of the 5000 most variable CpGs being available for further analysis. Several CNV events have previously been implicated in ccRCC aggressiveness and metastasis [1]; therefore, we examined whether these events are differentially distributed among 5mC-based clusters, which could further support the clinical and functional relevance of the observed clusters. The 4333 CpGs conserved across the two array platforms and CNV data calculated from SNP arrays in TCGA-KIRC for the same set of amplifications and deletions used in Fig. 6B were used for analysis. We observed that, as in Additional file 2: Fig. S16, two ccRCC clusters form, with cluster 1 enriched for M0 tumors and cluster 2 enriched for M1 tumors, and overall greater CNV enrichment in M1-enriched 5mC cluster 2 (Fig. 7A). CcRCCs in the M1 enriched cluster 2 are also significantly hypermethylated and carry more CNVs irrespective of their metastasis status (Figs. 7B, C). Furthermore, the M1 samples in cluster 1, despite the appearance of being ‘misclassified’, are significantly hypermethylated relative to M0’s in cluster 1 (not true for the M1 tumors in cluster 2, Fig. 7D). This suggests that M0 ccRCCs in cluster 2 are as hypermethylated as M1 tumors, despite not metastasizing, at least during the follow-up period available from TCGA.

Since the 4,333 CpGs discriminate between M0 and M1 primary ccRCC, suggestive of a 5mC signature of metastasis present in the primary tumor, we examined how they cluster ccRCC regions from our ITH analysis. We performed hierarchical clustering of the KIRC tumor samples with each tumor in our cohort (10–13 regions each) using the 4333 metastasis-associated CpGs (after performing batch correction), and identified tumor regions most epigenetically similar to and clustering with the KIRC M1 ccRCCs (not shown). Interestingly, the M1-like tumor regions from our ITH set are overall significantly more distant, in a phyloepigenetic tree, from normal kidney regions than M0-like tumor regions (*p* = 0.04; Fig. 7E). This finding is concordant with results in TCGA-KIRC where M1 tumors are also significantly farther from normal samples than M0 tumors (Additional file 2: Fig. S17). Furthermore, M1-like regions from our cohort carry significantly more CNV than M0-like tumor regions (*p* = 0.024; Fig. 7F). Finally, for one case (patient w3) we were able to obtain and analyze a single region from a matched synchronous pancreatic metastasis. In a phyloepigenetic tree drawn using the 4333 metastasis-associated CpGs, the pancreatic metastasis branches off from the same branch carrying two M1-like regions (Fig. 7G), providing further support that the 4333 CpGs do indeed flag regions within primary tumors with metastatic ‘tendency’. A similar phyloepigenetic tree branching pattern of the metastatic sample is observed when incorporated with the primary tumor regions of patient w3 and replotted using the previous 236 enhancer-associated CpGs (Fig. 7H compared with Additional file 2: Fig. S13). Figure 7H shows the gradual change in 5mC between normal samples and advanced tumor regions. The mean methylation of CpGs in two genes is hypomethylated in normal and hypermethylated at the terminal tumor regions (*USP44* and *SATB2*); the opposite is true for three other genes (*KRBA1*, *ANKS4B*, and *KDM8*). The magnitude of change is large and ranges between two- and sevenfold. Interestingly, of the 236 CpGs used to plot the tree in Fig. 7H, 22 are shared with the 4333 CpGs from Fig. 7G. Genes linked to these 22 CpGs include *USP44* (*n* = 2 CpGs), *KRBA1* (*n* = 2 CpGs), and *ANKS4B* (*n* = 1 CpG), suggesting that epigenetic deregulation of these loci plays a role in ccRCC pathogenesis. Taken together, these findings suggest that epigenetic level ITH provides information on the metastatic potential of individual regions within a tumor, which may, with further study, reveal prognostic signatures or novel epigenetic drivers of metastasis that could fill an important gap in our knowledge of this most insidious property of cancer.

## Discussion

Prior studies of ITH in ccRCC have focused on mutations or CNV; however, far less is known about epigenetic ITH. In the present work, we analyzed ITH at the DNA methylation level, correlated relationships with clinical and pathologic data, compared 5mC ITH to copy number ITH, examined the influence of *SETD2* mutation on ITH parameters, and finally applied our ITH data to better understand ccRCC metastatic potential. Our data reveal marked pathologic level heterogeneity within 5mC and H3K36me3 levels. While the latter generally correlates well with *SETD2* mutation status defined using a single region of the tumor, there are limits to the association, indicating that addition of H3K36me3 IHC to routine pathologic analysis would add value to mutation screening. Normal kidney shows modest levels of 5mC ITH, whereas ccRCC displays markedly elevated methylation variability that is highly enriched in kidney enhancers. We then linked 5mC variability to entropy and found that tumor 5mC entropy was strongly linked to clinical parameters including tumor grade and metastasis, and that *SETD2* wt tumors had greater 5mC entropy than *SETD2* mutant tumors. Standard differential methylation analysis revealed a dominating role for inter-patient variability within the methylation landscape, which necessitated use of intra-class correlation to pinpoint 5mC variability within and across individual patient tumor regions. This approach uncovered a set of CpGs with unique properties that robustly clustered tumor regions by clinical feature. Extending this approach, we applied phyloepigenetic analysis to uncover novel epigenetic drivers such as *USP44*, *ANKS4B*, and *KDM8*. Copy number was also analyzed revealing that higher CNV ITH correlated with lower global 5mC but higher 5mC entropy. Finally, we applied our ITH findings to a key aspect of ccRCC biology: understanding tumor metastatic potential. Our findings revealed that 5mC ITH data pinpoint epigenetic signatures related to metastasis.

Our study has several limitations. First, although we isolated small regions from individual slides, the approach is still a ‘bulk’ approach, and as such we are dealing with mixtures of cells. Although all the regions we analyzed were chosen to have > 60% tumor content, our results may nonetheless be influenced by cell composition. Advantages of the multi-region approach include much greater ‘coverage’ of the tumor, including coverage of a larger geographic area; a noted limitation of single-cell sequencing approaches. Second, at the DNA methylation level, single cell whole genome bisulfite sequencing is only just becoming available and suffers from low CpG coverage [46]. Third, we did not sequence each subregion within the tumor for loss of function mutations such as *VHL*, *BAP1*, and *PBRM1*. Given that *VHL* mutation is the only consistent truncal mutation in ccRCC, and nearly two-thirds of other ccRCC driver mutations are subclonal, a single region, single cell approach would almost certainly miss substantial ITH present at any level [1]. The importance of subclonal mutations to the tumor overall are only just beginning to be appreciated, but data suggest that they can drive overall tumor growth and contribute to development of new phenotypic traits [47]. Ideally, as single cell epigenome methods become more routine and their genome coverage increases, a future study addressing epigenetic ITH might combine the two approaches to achieve both macro- (i.e., regional) and micro- (single cell) level coverage to fully understand the functional consequences of ITH. As such, this would complement the work of Li et al*.* [48] who studied ccRCC heterogeneity using a multi-region analysis of scRNA-seq and exome sequencing mutational landscapes.

We initially hypothesized that loss of SETD2-mediated H3K36me3 and its ability to target 5mC via DNMT3B (and to a lesser extent DNMT3A [26]) would result in greater epigenetic disorder; however, we observed the opposite. This may indicate that loss of SETD2 is a strong driver of epigenome deregulation that directs tumor cells down a distinct pathway, whereas SETD2 proficient tumor cells sustain greater variability in the epigenome due to diversity of other genetic changes that may work only partly through the epigenome (e.g., VHL [49]) and/or epigenome-independent pathways. Our analysis also revealed that while there was a modest level of 5mC heterogeneity (or entropy) in the normal kidney, this was markedly elevated in ccRCC. Kidney 5mC variability was primarily enriched at gene bodies, whereas ccRCC 5mC variability was highly enriched in kidney enhancers. This suggests that variable CpGs in normal kidney are less involved with gene regulatory events (rather they may reflect other influences on gene expression such as genetic and/or environmental exposures), while the epigenetic heterogeneity within ccRCC has the potential to drive more dramatic effects on gene expression though changes in cell-type-specific enhancer function. Indeed, genes linked to normal kidney epigenetic heterogeneity are weakly enriched in specific pathways, including tumor-relevant pathways, whereas genes linked to enhancers subject to epigenome ITH are highly enriched in cancer-related pathways like epithelial-to-mesenchymal transition and CXCR4 signaling that may influence metastatic potential [49]. The importance of epigenetic ITH targeting enhancers was also evident from our finding that a small subset of enhancer 5mC changes linked to ITH within patient tumors robustly segregated tumor regions by clinically relevant properties including grade, stage, and metastasis, and uncovered both known and novel putative 5mC drivers in phyloepigenetic analyses. Thus, epigenetic ITH targeting enhancers could impact a diverse array of processes including cell survival and proliferation, response to chemotherapeutics, the tumor microenvironment, and metastatic potential. The implications of epigenetic heterogeneity, and differences driven by epigenetic regulator mutations like *SETD2* are likely relevant for both therapy and biomarker development. Use of phyloepigenetic approaches would enhance both efforts by aiding in identification of the most common/early epigenetic changes throughout the tumor, and for biomarker development since these truncal epigenetic changes may become targetable by future therapeutics. It is unclear how epigenetic heterogeneity will influence response to epigenetic drugs like 5-aza-2’-deoxycytidine, but, drawing inferences from genetic heterogeneity, less ITH may portend a better response to these agents since greater epigenetic heterogeneity would be expected to contribute to enhanced phenotypic heterogeneity upon which evolutionary selection can act [17]. Such analyses will be important as part of future studies to better understand how epigenetic-level ITH influences tumor properties and response to treatment.

During our analyses we noted that, for a number of measured parameters, there was a discrepancy between findings related to *SETD2* mutation status (defined by targeted exome sequencing of single region), and SETD2 ‘functionality’ defined by presence of H3K36me3 detected by IHC. For example, global methylation variability, Euclidian distances between tumor regions and normal kidney, and overall CNV number were higher in sequence-based *SETD2* wild-type tumors, but higher in IHC-based H3K36me3 negative tumor regions. In contrast, other parameters such as global 5mC and enhancer-based entropy were only significant for one of the two parameters. Given the high level of ITH reported within ccRCC at the mutational, copy number, immune microenvironment, and therapeutic response levels [1, 9, 10] this outcome may not be surprising. While on the one hand this could be discordance, it could also be because we did not sequence the *SETD2* gene in all tumor regions as this was not our main focus. Follow-up work performing targeted sequencing across *SETD2* might reveal the presence of subclonal mutations, which are common in ccRCC [1]. While a number of reports, including from our own group, have used H3K36me3 as a surrogate for *SETD2* mutational status due to the lack of suitable IHC grade SETD2 antibodies [25, 28], in most cases only the single most clinically aggressive region is examined by sequencing. Discordance between methodologies used to assess the mutational status of other oncogenes and tumor suppressor genes has been noted previously, including *HER2* amplification and overexpression in breast cancer [50], *TP53* mutations in ovarian cancer [51], *BRAF* V600E mutation status in colorectal cancer [52], and aberrant cytoplasmic localization of nucleophosmin in acute myeloid leukemia [53]. While *SETD2* mutation may initiate loss of H3K36me3, *SETD2* mutant cells initially may comprise only a small fraction of the tumor mass, and/or be distributed non-uniformly throughout the tumor. Whether this immediately leads to H3K36me3 loss or whether other factors might initially compensate, such as H3K36me2 driven by the NSD1/2/3 family, is unknown. Indeed, when SETD2 is knocked out in mouse models, the H3K36me2 mark partially redistributes to regions formerly enriched for H3K36me3 [26] and in vitro studies show that NSD factors can perform mono, di, and trimethylation at the H3K36 position [54]. Alternatively, other methods of gene inactivation, such as epigenetic silencing though promoter methylation, may contribute to the observed discordance. *VHL*, for example, is intact but silenced by DNA hypermethylation in 11–35% of ccRCC cases [55, 56]. While we did not find evidence for *SETD2* promoter hypermethylation in this study (data not shown, consistent with [57]), other regulators of H3K36 methylation like NSD1 can be targeted in this way in ccRCC [58]. An additional issue relates to the nature of the *SETD2* mutations themselves. While most of the *SETD2* mutations in our cohort are likely inactivating because they are frameshift or nonsense mutations (5/6), one patient’s tumor contained a missense mutation (R1592P in patient m11) that is predicted as ‘probably damaging’ by Polyphen-2. Indeed, this patient showed 7 out 10 regions positive for H3K36me3 by IHC. Given SETD2’s large size, very few cancer mutations have been functionally studied for their effect on enzymatic activity, DNA binding, subcellular localization, or protein stability. We are not aware of a systematic intratumor comparison of these two parameters for ccRCC outside of our study. Our results indicate that more comprehensive co-analyses of *SETD2* mutation and H3K36me3 levels are warranted in future studies, and will likely be essential to fully understand their interplay with tumor phenotypes and patient outcome.

## Conclusions

This study represents, to our knowledge, the first investigation into 5mC ITH in ccRCC using a multi-region sampling approach. Our results reveal marked ITH at the epigenome level, particularly targeting kidney enhancers. Furthermore, using epigenetic ITH findings we identified a 5mC signature that links primary tumor regions with more aggressive clinical characteristics to metastatic potential.

## Methods

Upon institutional review board approval, patients who underwent radical nephrectomy for unilateral ccRCC were identified through the Mayo Clinic Biobank. FFPE blocks containing predominantly histologically viable-appearing tumor cells were identified by a genitourinary pathologist (MLS). Sections from each block were cut and mounted onto glass slides, and then each slide was further divided into 3–6 regions for DNA isolation (typically the same region was cut and pooled from 3 to 5 adjacent re-cuts to obtain sufficient DNA, Fig. 1). This yielded 10–13 separate tumor regions from each patient (138 tumor regions total from 12 patients). DNA was extracted and genomic mutational profiles determined using a targeted next generation sequencing cancer gene panel. From these samples, we identified six *SETD2* wild-type (wt) and six *SETD2* mutant (mt) cases to allow us to compare the impact of this mutation, known to regulate H3K36me3 and 5mC patterns [24] on epigenome-level ITH. Clinical and pathologic characteristics of each tumor are summarized in Table 1 and listed individually by patient, along with their gene mutation profiles from the targeted gene sequencing panel in Additional file 1: Tables S1 and S3. We also obtained three snap-frozen non-cancerous kidney tissue samples from the National Disease Research Interchange (NRDI). Information on the normal kidney samples is also listed in Table 1 and Additional file 1: Table S5.

5mC profiling from normal kidney and ccRCC regions was measured using the Infinium MethylationEPIC array (Illumina) run at the University of Minnesota Genomics Core Facility. IDAT files were preprocessed using the commonly used normalization [59] and QC pipelines [60] before obtaining methylation β values.

All analyses such as differential methylation, copy number variation (CNV), intraclass correlation coefficient (ICC), singular value decomposition (SVD), entropy, and Euclidian distances measurement, as well as plot generation were executed in an R environment (version 3.6.2). Ingenuity Pathway Analysis (IPA, Qiagen) and Genomic Regions Enrichments of Annotation Tool (GREAT) [61] were used for gene ontology and comparative analyses. Additional details regarding these methodologies can be found in the Supplemental Methods section in Additional file 2.

## Supplementary Information

**Additional file 1. Table S1**. Detailed summary of clinical parameters and patient data in ccRCC cohort.** Table S2**. Summary of ccRCC patients, number of FFPE blocks, and number of studied regions per block.** Table S3**. Detailed summary of mutations for each ccRCC patient.** Table S4**. List of 8,749 differentially methylated CpGs between ccRCC tumors and normals. Data are provided as a subset of Illumina EPIC array.** Table S5**. Detailed summary of medical history and patient data in normal kidney cohort.** Table S6**. List of 5,000 most variable CpGs in the intermediate ICC group. Data are provided as a subset of Illumina EPIC array.** Table S7**. List of 819 CpGs in the intermediate ICC groupassociated with survival in the TCGA-KIRC dataset. Data are provided as a subset of Illumina 450K array.** Table S8**. List of 435 GeneHancer CpGs conserved in at least any combination of 4 tumors. Data are provided as a subset of Illumina EPIC array.** Table S9**. List of 236 GeneHancer CpGs interacting with the gene they are located in. Data are provided as a subset of Illumina EPIC array.** Table S10**. List of 49 GeneHancer CpGs interacting with the gene they are located in and associated with better survival. Data are provided as a subset of Illumina 450K array.** Table S11**. Summary of Gerlinger and Gulati CNVs in ccRCC regions for each patient.

**Additional file 2. Fig. S1**. Comparison of averaged normal, wt, and mt feature methylation.** Fig. S2**. DMCpGs counts at various Δβ cutoffs.** Fig. S3**. *PRDM16* browser view and survival analysis.** Fig. S4**.* DPP6* browser view and survival analysis.** Fig. S5**. Comparison of three normal kidneys feature methylation.** Fig. S6**. Comparison of three normal kidneys feature entropy.** Fig. S7**. Entropy and clinico-pathological traits.** Fig. S8**. Boxplot showing methylation of single CpG in different regions of tumors.** Fig. S9**. Exploratory analysis of CpGs based on ICC grouping.** Fig. S10**. Supervised hierarchical clustering for low and high ICC lists of CpGs.** Fig. S11**. Flowchart denoting relationships between the 267,991 CpGs in the ICC 0.4 - 0.6 group and survival in TCGA-KIRC.** Fig. S12**. Analysis of highly ranked GeneHancer CpGs following SVD.** Fig. S13**. Phyloepigenetic trees and ITH driver genes.** Fig. S14**. Comparison of gene expression survival outcomes for drivers of ITH.** Fig. S15**. ITH at the CNV level and its relationship with clinical and pathologic parameters.** Fig. S16**. Unsupervised hierarchical clustering highlighting M0 and M1 segregation in TCGA-KIRC.** Fig. S17**. Contrast of M0 and M1 Euclidian distances from adjacent normal samples in TCGA-KIRC.

## Data Availability

DNA methylation data generated in this study are available for download in NCBI GEO accession GSE206049.

## Abbreviations

5mC: 5-Methylcytosine
CNV: Copy number variation
ccRCC: Clear cell renal cell carcinoma
DMCpG: Differentially methylated CpG
EMT: Epithelial-to-mesenchymal transition
ENCODE: Encyclopedia of DNA elements
FFPE: Formalin-fixed paraffin-embedded
GREAT: Genomic Regions Enrichments of Annotation Tool
ICC: Intra-class correlation coefficient
IHC: Immunohistochemistry
IPA: Ingenuity Pathway Analysis
ITH: Intra-tumor heterogeneity
mt: Mutant
NDRI: National Disease Research Interchange
PCA: Principal component analysis
RCC: Renal cell carcinoma
scRNA-seq: Single cell RNA sequencing
SSIGN: Stage, size, grade, and necrosis
SVD: Singular value decomposition
TCGA-KIRC: The cancer genome atlas kidney renal cell carcinoma
wt: Wild type

## References

[1] Gerlinger M, Horswell S, Larkin J, Rowan AJ, Salm MP, Varela I (2014). Genomic architecture and evolution of clear cell renal cell carcinomas defined by multiregion sequencing. Nat Genet.

[2] Choueiri TK, Tomczak P, Park SH, Venugopal B, Ferguson T, Chang Y-H (2021). Adjuvant pembrolizumab after nephrectomy in renal-cell carcinoma. N Engl J Med.

[3] Chakravarty D, Johnson A, Sklar J, Lindeman NI, Moore K, Ganesan S (2022). Somatic genomic testing in patients with metastatic or advanced cancer: ASCO provisional clinical opinion. J Clin Oncol.

[4] Motzer R, Alekseev B, Rha S-Y, Porta C, Eto M, Powles T (2021). Lenvatinib plus pembrolizumab or everolimus for advanced renal cell carcinoma. N Engl J Med.

[5] Choueiri TK, Powles T, Burotto M, Escudier B, Bourlon MT, Zurawski B (2021). Nivolumab plus cabozantinib versus sunitinib for advanced renal-cell carcinoma. N Engl J Med.

[6] Rini BI, Plimack ER, Stus V, Gafanov R, Hawkins R, Nosov D (2019). Pembrolizumab plus axitinib versus sunitinib for advanced renal-cell carcinoma. N Engl J Med.

[7] Linehan WM, Ricketts CJ (2019). The Cancer Genome Atlas of renal cell carcinoma: findings and clinical implications. Nat Rev Urol.

[8] Nowell PC (1976). The clonal evolution of tumor cell populations. Science (80-).

[9] Martinez P, Birkbak NJ, Gerlinger M, McGranahan N, Burrell RA, Rowan AJ (2013). Parallel evolution of tumour subclones mimics diversity between tumours. J Pathol.

[10] Crusz SM, Tang YZ, Sarker SJ, Prevoo W, Kiyani I, Beltran L (2016). Heterogeneous response and progression patterns reveal phenotypic heterogeneity of tyrosine kinase inhibitor response in metastatic renal cell carcinoma. BMC Med..

[11] Gerlinger M, Rowan AJ, Horswell S, Larkin J, Endesfelder D, Gronroos E (2012). Intratumor heterogeneity and branched evolution revealed by multiregion sequencing. N Engl J Med.

[12] Jonasch E, Donskov F, Iliopoulos O, Rathmell WK, Narayan VK, Maughan BL (2021). Belzutifan for renal cell carcinoma in von Hippel–Lindau Disease. N Engl J Med.

[13] Tiedemann RL, Putiri EL, Lee JH, Hlady RA, Kashiwagi K, Ordog T (2014). Acute depletion redefines the division of labor among DNA methyltransferases in methylating the human genome. Cell Rep.

[14] Ricketts CJ, De Cubas AA, Fan H, Smith CC, Lang M, Reznik E (2018). The Cancer Genome Atlas comprehensive molecular characterization of renal cell carcinoma. Cell Rep.

[15] El Khoury LY, De Fu S, Hlady RA, Wagner RT, Wang L, Eckel-Passow JE (2021). Identification of DNA methylation signatures associated with poor outcome in lower-risk Stage, Size, Grade and Necrosis (SSIGN) score clear cell renal cell cancer. Clin Epigenetics..

[16] Flavahan WA, Gaskell E, Bernstein BE (2021). Epigenetic plasticity and the hallmarks of cancer. Science.

[17] Marusyk A, Janiszewska M, Polyak K (2020). Intratumor heterogeneity: the rosetta stone of therapy resistance. Cancer Cell.

[18] Chaligne R, Gaiti F, Silverbush D, Schiffman JS, Weisman HR, Kluegel L (2021). Epigenetic encoding, heritability and plasticity of glioma transcriptional cell states. Nat Genet.

[19] Hansen KD, Timp W, Bravo HC, Sabunciyan S, Langmead B, McDonald OG (2011). Increased methylation variation in epigenetic domains across cancer types. Nat Genet.

[20] Baylin SB, Jones PA (2011). A decade of exploring the cancer epigenome - biological and translational implications. Nat Rev Cancer.

[21] Gunasekara CJ, Scott CA, Laritsky E, Baker MS, MacKay H, Duryea H, Jones JD (2019). A genomic atlas of systemic interindividual epigenetic variation in humans. Genome Biol..

[22] Turajlic S, Xu H, Litchfield K, Rowan A, Chambers T, Lopez JI (2018). Tracking cancer evolution reveals constrained routes to metastases: TRACERx renal. Cell.

[23] Ho TH, Choueiri TK, Wang K, Karam JA, Chalmers Z, Frampton G (2016). Correlation between molecular subclassifications of clear cell renal cell carcinoma and targeted therapy response. Eur Urol Focus.

[24] Tiedemann RL, Hlady RA, Hanavan PD, Lake DF, Tibes R, Lee JH (2015). Dynamic reprogramming of DNA methylation in SETD2-deregulated renal cell carcinoma. Oncotarget.

[25] Ho TH, Kapur P, Joseph RW, Serie DJ, Eckel-Passow JE, Tong P (2016). Loss of histone H3 lysine 36 trimethylation is associated with an increased risk of renal cell carcinoma-specific death. Mod Pathol.

[26] Weinberg DN, Papillon-Cavanagh S, Chen H, Yue Y, Chen X, Rajagopalan KN (2019). The histone mark H3K36me2 recruits DNMT3A and shapes the intergenic DNA methylation landscape. Nature.

[27] Xie Y, Sahin M, Sinha S, Wang Y, Nargund AM, Lyu Y (2022). SETD2 loss perturbs the kidney cancer epigenetic landscape to promote metastasis and engenders actionable dependencies on histone chaperone complexes. Nat Cancer..

[28] Bihr S, Ohashi R, Moore AL, Rüschoff JH, Beisel C, Hermanns T (2019). Expression and mutation patterns of PBRM1, BAP1 and SETD2 mirror specific evolutionary subtypes in clear cell renal cell carcinoma. Neoplasia (United States)..

[29] Kang HW, Park H, Seo SP, Byun YJ, Piao XM, Kim SM (2019). Methylation signature for prediction of progression free survival in surgically treated clear cell renal cell carcinoma. J Korean Med Sci..

[30] Glazko G, Mushegian A (2010). Measuring gene expression divergence: The distance to keep. Biol Direct.

[31] Hannum G, Guinney J, Zhao L, Zhang L, Hughes G, Sadda S (2013). Genome-wide methylation profiles reveal quantitative views of human aging rates. Mol Cell.

[32] Suo F, Pan M, Li Y, Yan Q, Hu H, Hou L (2021). Mesenchymal stem cells cultured in 3D system inhibit non-small cell lung cancer cells through p38 MAPK and CXCR4/AKT pathways by IL-24 regulating. Mol Biol.

[33] Yehia L, Keel E, Eng C (2020). The Clinical Spectrum of PTEN Mutations. Annu Rev Med..

[34] Planterose Jiménez B, Liu F, Caliebe A, Montiel González D, Bell JT, Kayser M (2021). Equivalent DNA methylation variation between monozygotic co-twins and unrelated individuals reveals universal epigenetic inter-individual dissimilarity. Genome Biol..

[35] Bose M, Wu C, Pankow JS, Demerath EW, Bressler J, Fornage M (2014). Evaluation of microarray-based DNA methylation measurement using technical replicates: The atherosclerosis risk in communities (ARIC) study. BMC Bioinformatics..

[36] Kundu A, Nam H, Shelar S, Chandrashekar DS, Brinkley G, Karki S (2020). PRDM16 suppresses HIF-targeted gene expression in kidney cancer. J Exp Med..

[37] Zhang Z, Lin E, Zhuang H, Xie L, Feng X, Liu J (2020). Construction of a novel gene-based model for prognosis prediction of clear cell renal cell carcinoma. Cancer Cell Int.

[38] Fishilevich S, Nudel R, Rappaport N, Hadar R, Plaschkes I, Stein TI (2017). GeneHancer: Genome-wide integration of enhancers and target genes in GeneCards. Database.

[39] Zhou J, Wang T, Qiu T, Chen Z, Ma X, Zhang L (2020). Ubiquitin-specific protease-44 inhibits the proliferation and migration of cells via inhibition of JNK pathway in clear cell renal cell carcinoma. BMC Cancer.

[40] Dentro SC, Leshchiner I, Haase K, Tarabichi M, Wintersinger J, Deshwar AG (2021). Characterizing genetic intra-tumor heterogeneity across 2658 human cancer genomes. Cell.

[41] Gao Y, Widschwendter M, Teschendorff AE (2018). DNA methylation patterns in normal tissue correlate more strongly with breast cancer status than copy-number variants. EBioMedicine.

[42] Shi X, Radhakrishnan S, Wen J, Chen JY, Chen J, Lam BA (2020). Association of CNVs with methylation variation. Npj Genomic Med.

[43] Feber A, Guilhamon P, Lechner M, Fenton T, Wilson G, Thirlwell C (2014). Using high-density DNA methylation arrays to profile copy number alterations. Genome Biol.

[44] Gulati S, Martinez P, Joshi T, Birkbak NJ, Santos CR, Rowan AJ (2014). Systematic evaluation of the prognostic impact and intratumour heterogeneity of clear cell renal cell carcinoma biomarkers. Eur Urol.

[45] Hua X, Zhao W, Pesatori AC, Consonni D, Caporaso NE, Zhang T (2020). Genetic and epigenetic intratumor heterogeneity impacts prognosis of lung adenocarcinoma. Nat Commun.

[46] Smallwood SA, Lee HJ, Angermueller C, Krueger F, Saadeh H, Peat J (2014). Single-cell genome-wide bisulfite sequencing for assessing epigenetic heterogeneity. Nat Methods.

[47] Marusyk A, Tabassum DP, Altrock PM, Almendro V, Michor F, Polyak K (2014). Non-cell-autonomous driving of tumour growth supports sub-clonal heterogeneity. Nature.

[48] Li R, Ferdinand JR, Loudon KW, Bowyer GS, Laidlaw S, Muyas F (2022). Mapping single-cell transcriptomes in the intra-tumoral and associated territories of kidney cancer. Cancer Cell.

[49] Vanharanta S, Shu W, Brenet F, Hakimi AA, Heguy A, Viale A (2012). Epigenetic expansion of VHL-HIF signal output drives multiorgan metastasis in renal cancer. Nat Med.

[50] Memon R, Prieto Granada CN, Harada S, Winokur T, Reddy V, Kahn AG (2022). Discordance between immunohistochemistry and in situ hybridization to detect HER2 Overexpression/Gene amplification in breast cancer in the modern age: a single institution experience and pooled literature review study: Discordance between HER2 overexpression and gene amplification in breast cancer. Clin Breast Cancer.

[51] Kang EY, Cheasley D, LePage C, Wakefield MJ, da Cunha TM, Rowley S (2021). Refined cut-off for TP53 immunohistochemistry improves prediction of TP53 mutation status in ovarian mucinous tumors: implications for outcome analyses. Mod Pathol.

[52] Estrella JS, Tetzlaff MT, Bassett RL, Patel KP, Williams MD, Curry JL (2015). Assessment of BRAF V600E status in colorectal carcinoma: tissue-specific discordances between immunohistochemistry and sequencing. Mol Cancer Ther.

[53] Konoplev S, Xuelin H, Drabkin HA, Koeppen H, Jones D, Kantarjian HM (2009). Cytoplasmic localization of nucleophosmin in bone marrow blasts of acute myeloid leukemia patients is not completely concordant with NPM1 mutation and is not predictive of prognosis. Cancer.

[54] Morishita M, Mevius D, di Luccio E (2014). In vitro histone lysine methylation by NSD1, NSD2/MMSET/WHSC1, and NSD3/WHSC1L. BMC Struct Biol.

[55] Cowey CL, Rathmell WK (2009). VHL gene mutations in renal cell carcinoma: Role as a biomarker of disease outcome and drug efficacy. Curr Oncol Rep.

[56] Khaliq S, Ajaz S, Firasat S, Shahid S, Hasan AS, Sultan G (2014). Unique molecular alteration patterns in von Hippel-Lindau (VHL) gene in a cohort of sporadic renal cell carcinoma patients from Pakistan. Mutat Res Fundam Mol Mech Mutagen.

[57] Ibragimova I, Maradeo ME, Dulaimi E, Cairns P (2013). Aberrant promoter hypermethylation of PBRM1, BAP1, SETD2, KDM6A and other chromatin-modifying genes is absent or rare in clear cell RCC. Epigenetics.

[58] Su X, Zhang J, Mouawad R, Compérat E, Rouprêt M, Allanic F (2017). NSD1 inactivation and SETD2 mutation drive a convergence toward loss of function of H3K36 writers in clear cell renal cell carcinomas. Cancer Res.

[59] Aryee MJ, Jaffe AE, Corrada-Bravo H, Ladd-Acosta C, Feinberg AP, Hansen KD (2014). Minfi: a flexible and comprehensive Bioconductor package for the analysis of Infinium DNA methylation microarrays. Bioinformatics.

[60] Pidsley R, Y Wong CC, Volta M, Lunnon K, Mill J, Schalkwyk LC (2013). A data-driven approach to preprocessing Illumina 450K methylation array data. BMC Genomics.

[61] McLean CY, Bristor D, Hiller M, Clarke SL, Schaar BT, Lowe CB (2010). GREAT improves functional interpretation of cis-regulatory regions. Nat Biotechnol.

