# Supplementary material for: Extensive intratumor regional epigenetic heterogeneity in clear cell renal cell carcinoma targets kidney enhancers and is associated with poor outcome
Source: Clin Epigenetics. 2023 Apr 29;15:71. doi: 10.1186/s13148-023-01471-3 (PMC10149001; doi:10.1186/s13148-023-01471-3)
Supplement: Supplementary file 2 — Additional file 2. Fig. S1. Comparison of averaged normal, wt, and mt feature methylation. Fig. S2. DMCpGs counts at various Δβ cutoffs. Fig. S3. PRDM16 browser view and survival analysis. Fig. S4. DPP6 browser view and survival analysis. Fig. S5. Comparison of three normal kidneys feature methylation. Fig. S6. Comparison of three normal kidneys feature entropy. Fig. S7. Entropy and clinico-pathological traits. Fig. S8. Boxplot showing methylation of single CpG in different regions of tumors. Fig. S9. Exploratory analysis of CpGs based on ICC grouping. Fig. S10. Supervised hierarchical clustering for low and high ICC lists of CpGs. Fig. S11. Flowchart denoting relationships between the 267,991 CpGs in the ICC 0.4 - 0.6 group and survival in TCGA-KIRC. Fig. S12. Analysis of highly ranked GeneHancer CpGs following SVD. Fig. S13. Phyloepigenetic trees and ITH driver genes. Fig. S14. Comparison of gene expression survival outcomes for drivers of ITH. Fig. S15. ITH at the CNV level and its relationship with clinical and pathologic parameters. Fig. S16. Unsupervised hierarchical clustering highlighting M0 and M1 segregation in TCGA-KIRC. Fig. S17. Contrast of M0 and M1 Euclidian distances from adjacent normal samples in TCGA-KIRC. [file 13148_2023_1471_MOESM2_ESM.docx]

**Supplemental material for El Khoury *et al*., Extensive intratumor regional epigenetic heterogeneity in clear cell renal cell carcinoma targets kidney enhancers and is associated with poor outcome**

**Supplemental Methods**

Clinical samples and clinical/pathological features/mutation sequencing

Following institutional review board approval, the Mayo Clinic Biobank was queried to identify patients who underwent radical nephrectomy for unilateral ccRCC between the years 2007 and 2019. FFPE blocks containing predominantly histologically viable-appearing tumor cells with >60% tumor nuclei and <20% necrosis by sample volume were identified by a genitourinary pathologist (MLS, using criteria established by TCGA). For ccRCC samples available as multiple FFPE blocks corresponding to distinct regions of the tumor, DNA was extracted from the region with the highest grade, meeting TCGA criteria [1], using the AllPrep DNA/RNA FFPE Kit (Qiagen), and subjected to genomic mutational profiling with a targeted next generation sequencing cancer gene panel. The panel was comprised of 600 established cancer-related genes and was performed on hybridization-captured, adaptor ligation-based libraries in a CLIA-certified laboratory, to a median sequencing depth of at least 650x [2]. Sequencing data was examined for base substitutions, short insertions, deletions, gene fusions, rearrangements, and copy number variation using Bayesian algorithms to call substitutions and local assembly algorithms to detect insertions, deletions, and copy number variation relative to a normal kidney control [2]. From these samples, we identified six *SETD2* wild-type (wt) and six *SETD2* mutant (mt) cases to allow us to compare the impact of this mutation, known to regulate H3K36me3 and DNA methylation patterns [3], on epigenome-level ITH. Clinical and pathologic characteristics of each tumor are summarized in Table 1 and listed individually by patient, along with their gene mutation profiles from the targeted gene sequencing panel in **Additional File 1: Table S1**. Each patient’s tumor was represented in at least five spatially distinct FFPE blocks. From each block multiple sections were cut (10-micron thickness), mounted onto glass slides, then each slide was further divided into 3-6 regions for DNA isolation (typically the same region was cut and pooled from 3-5 adjacent re-cuts to obtain sufficient DNA), as shown schematically in **Fig. 1**. This yielded 10-13 separate tumor regions from each patient (138 tumor regions total from 12 patients). A single ccRCC synchronous metastatic sample from the pancreas of patient w3 was also obtained. Adjacent recuts were also stained with H&E for pathologic evaluation and used for immunohistochemical staining with H3K36me3 (Abcam ab9050) and DNA methylation (5mC, Calbiochem/Sigma NA81) antibodies, as we have done previously [4,5]. DNA was extracted using the same method described above and was stored for downstream analysis. To fully understand ccRCC heterogeneity it was important to understand heterogeneity within normal, cancer-free, kidney as a reference. Therefore, we obtained three snap-frozen non-cancerous kidney tissue samples from the National Disease Research Interchange (NRDI) and divided each into ten regions for DNA isolation (using the DNeasy Blood & Tissue Kit, Qiagen) and analysis. Information on the normal kidney samples is also listed in Table 1 and **Additional File 1: Table S2**

DNA methylation analysis by Infinium MethylationEPIC array

DNA from each of the subdivided normal kidney and ccRCC regions was quantified using a Qubit fluorimeter then subjected to genome-wide DNA methylation profiling using the Infinium MethylationEPIC array (Illumina) run at the University of Minnesota Genomics Core Facility. IDAT files were preprocessed using the pipeline in the *minfi* (v 1.36.0) Bioconductor package [6] and normalized using the *minfi::swan* function before obtaining methylation β values. After the removal of probes with low detection scores, SNPs, and CpGs on sex chromosomes, the final β matrix was made of 843,393 CpGs. Samples with > 10% failing CpGs, those flagged by the *wateRmelon::outlyx* function as outliers, and those with a calculated bisulfite conversion score <80% using the *wateRmelon::bscon* function, were excluded from further analysis [7].

Copy number variation (CNV) analysis by Infinium MethylationEPIC array

CNV analysis based on Illumina Methylation EPIC array data was conducted using the *conumee* Bioconductor package [8], which has been established as the most reliable tool for EPIC array CNV calling [9]. Raw methylation data (IDAT files) from our cohort (normal n =30, ccRCC n=138) were preprocessed using the *minfi* Bioconductor package pipeline and copy number signal intensities for ccRCC samples were then called, relative to the normal samples, for predefined genomic bins using *conumee*. CNV gains and losses were called using established signal intensity cutoffs (log2 = ±0.1) [10].

Statistical analysis

All analyses were executed in an R environment (version 3.6.2). DNA methylation differences for each CpG site between normal and tumor groups, or between the *SETD2* wt and mt ccRCC groups were assessed using the *CpGassoc::cpg.assoc* function [11]. The criteria for differential methylation included p < 0.01 and a change in methylation of at least 10% (|Δβ_(tumor-normal)_| ≥ 0.10) [12]. The R package *ggpubr* was used to plot boxplots and scatter plots. Phylogenetic/epigenetic trees were constructed using the R packages *ape* and *ggtree,* and heatmaps were constructed using R packages *heatmap3* and *gplots*. Browser views were generated using the *Gviz* package [13]. Ingenuity Pathway Analysis (IPA, Qiagen) and Genomic Regions Enrichments of Annotation Tool (GREAT) [14] were used for gene ontology and comparative analyses. Linear relationships between DNA methylation and gene expression in TCGA data were assessed using Pearson correlation and survival analysis between CpGs in Illumina 450k array and TCGA samples were obtained from the methsurv database [15].

To ensure the selection of CpGs pertaining to variation “within” and “between” tumors, we calculated the intraclass correlation coefficient (ICC) [16,17]. For each CpG in the bundle of samples belonging to the same tumor, ICC was calculated using the *JWileymisc::iccMixed* R function. An ICC with a value close to 0 indicates that a CpG’s variation is driven by patient-specific elements (intra-tumor differences), whereas an ICC close to 1 indicates that a CpG’s variation is driven by population-wide differences (inter-tumor differences). We opted for selecting the group of CpGs with intermediate ICC (0.4 – 0.6) for downstream analysis as it is not biased towards intra- or inter-tumor differences.

To identify CpGs that most influence variance within each set of tumor samples, we performed singular value decomposition (SVD) [18]. SVD summarizes the main ways mean-centered data deviate from zero, allowing us to identify loci likely to be important in driving a phenotype of interest. The first step of an SVD analysis is the mean-centering of the beta values which was performed using the *sweep* function in R. The second step is a principal component analysis (PCA) of the newly acquired mean-centered beta matrix using the *prcomp* R function. Finally the variance for principal component 1 was extracted using the *factoextra::get_pca_var* R function.

Determining regions of primary ccRCC with similarity to metastatic tumors

CpGs with discriminatory potential between M0 and M1 ccRCCs from TCGA-KIRC were identified using unsupervised hierarchical clustering. Of the 5,000 most variable CpGs, 4,333 are shared between TCGA-KIRC (analyzed using 450K array) and our cohort (analyzed using the EPIC array). To determine the proximity of the samples in our cohort to either M0 or M1 tumors, we performed 12 independent hierarchical clustering analyses. In each of the 12 analyses we include TCGA-KIRC samples and all samples originating from the same tumor. For each of the tumors in our cohort, a separate batch correction is conducted using the *sva::ComBat* R function [19]. The regions from our cohort that cluster with the TCGA-KIRC M0 tumors are labelled “M0-like” and those clustering with TCGA-KIRC M1 are labelled as “M1-like” tumors.

Published dataset use

To cross reference our findings with a second cohort we acquired genomic (CNV and gene expression) and epigenomic (DNA methylation) data of TCGA-KIRC from the UCSC Xena Browser [20]. When it was necessary to combine DNA methylation data from our cohort and KIRC, we performed batch correction using the *sva::ComBat* R function [19].

To establish an enhancer landscape for normal kidney, we used publicly available data from Encyclopedia of DNA Elements (ENCODE) for histone marks H3K27ac, H3K4me1, and H3K4me3 with accession numbers GSM1112799, GSM773001, and GSM773005, respectively. These marks were acquired from the kidney of the same 50-year-old male. Across the genome, regions with overlapping H3K27ac, H3K4me1, but not H3K4me3, were considered active enhancers.

References

1. Ho TH, Nateras RN, Yan H, Park JG, Jensen S, Borges C, et al. A Multidisciplinary Biospecimen Bank of Renal Cell Carcinomas Compatible with Discovery Platforms at Mayo Clinic, Scottsdale, Arizona. PLoS One. Public Library of Science; 2015;10:e0132831.

2. Ho TH, Choueiri TK, Wang K, Karam JA, Chalmers Z, Frampton G, et al. Correlation Between Molecular Subclassifications of Clear Cell Renal Cell Carcinoma and Targeted Therapy Response. Eur Urol Focus. 2016;2.

3. Tiedemann RL, Hlady RA, Hanavan PD, Lake DF, Tibes R, Lee JH, et al. Dynamic reprogramming of DNA methylation in SETD2-deregulated renal cell carcinoma. Oncotarget. 2015/12/10. 2015;7:1927–46.

4. Ho TH, Kapur P, Joseph RW, Serie DJ, Eckel-Passow JE, Tong P, et al. Loss of histone H3 lysine 36 trimethylation is associated with an increased risk of renal cell carcinoma-specific death. Mod Pathol. United States & Canadian Academy of Pathology; 2016;29:34–42.

5. Ho TH, Park IY, Zhao H, Tong P, Champion MD, Yan H, et al. High-resolution profiling of histone h3 lysine 36 trimethylation in metastatic renal cell carcinoma. Oncogene. Macmillan Publishers Limited; 2016;35:1565–74.

6. Aryee MJ, Jaffe AE, Corrada-Bravo H, Ladd-Acosta C, Feinberg AP, Hansen KD, et al. Minfi: a flexible and comprehensive Bioconductor package for the analysis of Infinium DNA methylation microarrays. Bioinformatics. 2014;30.

7. Pidsley R, Y Wong CC, Volta M, Lunnon K, Mill J, Schalkwyk LC. A data-driven approach to preprocessing Illumina 450K methylation array data. BMC Genomics. 2013;14:293.

8. Hovestadt V, Zapatka M. Conumee: Enhanced copy-number variation analysis using Illumina DNA methylation arrays. 2017;

9. Kilaru V, Knight AK, Katrinli S, Cobb D, Lori A, Gillespie CF, et al. Critical evaluation of copy number variant calling methods using DNA methylation. Genet Epidemiol. 2020;44.

10. Gao Y, Widschwendter M, Teschendorff AE. DNA Methylation Patterns in Normal Tissue Correlate more Strongly with Breast Cancer Status than Copy-Number Variants. EBioMedicine. 2018;

11. Barfield RT, Kilaru V, Smith AK, Conneely KN. CpGassoc: An R function for analysis of DNA methylation microarray data. Bioinformatics. 2012;

12. Yang M, Hlady RA, Zhou D, Ho TH, Robertson KD. In silico DNA methylation analysis identifies potential prognostic biomarkers in type 2 papillary renal cell carcinoma. Cancer Med. 2019;

13. Hahne F, Ivanek R. Visualizing Genomic Data Using Gviz and Bioconductor. Humana Press, New York, NY; 2016. p. 335–51.

14. McLean CY, Bristor D, Hiller M, Clarke SL, Schaar BT, Lowe CB, et al. GREAT improves functional interpretation of cis-regulatory regions. Nat Biotechnol. Nature Publishing Group; 2010;28:495–501.

15. Modhukur V, Iljasenko T, Metsalu T, Lokk K, Laisk-Podar T, Vilo J. MethSurv: a web tool to perform multivariable survival analysis using DNA methylation data. Epigenomics. 2018;10:277–88.

16. Planterose Jiménez B, Liu F, Caliebe A, Montiel González D, Bell JT, Kayser M, et al. Equivalent DNA methylation variation between monozygotic co-twins and unrelated individuals reveals universal epigenetic inter-individual dissimilarity. Genome Biol. 2021;22.

17. Bose M, Wu C, Pankow JS, Demerath EW, Bressler J, Fornage M, et al. Evaluation of microarray-based DNA methylation measurement using technical replicates: The atherosclerosis risk in communities (ARIC) study. BMC Bioinformatics. 2014;15.

18. Mazor T, Pankov A, Johnson BE, Hong C, Hamilton EG, Bell RJA, et al. DNA methylation and somatic mutations converge on the cell cycle and define similar evolutionary histories in brain tumors. Cancer Cell. 2015;28:307–17.

19. Leek JT, Johnson WE, Parker HS, Jaffe AE, Storey JD. The sva package for removing batch effects and other unwanted variation in high-throughput experiments. Bioinformatics. 2012/01/20. 2012;28:882–3.

20. Goldman MJ, Craft B, Hastie M, Repečka K, McDade F, Kamath A, et al. Visualizing and interpreting cancer genomics data via the Xena platform. Nat. Biotechnol. 2020.

**Supplemental figures and figure legends for El Khoury *et al*., Extensive intratumor regional epigenetic heterogeneity in clear cell renal cell carcinoma targets kidney enhancers and is associated with poor outcome**


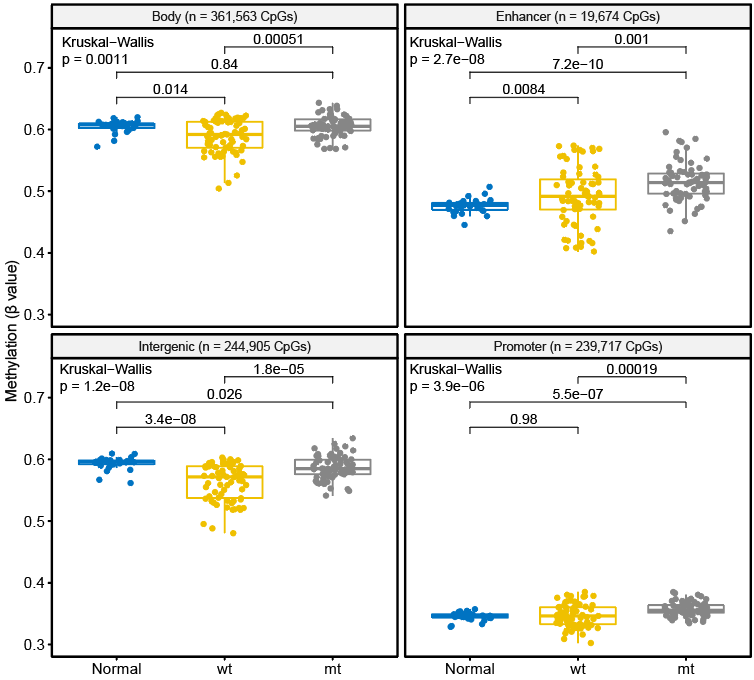


**Supplemental Figure S1.** Boxplots showing feature-based methylation levels for normal kidney as well as *SETD2* wt and mt ccRCC from our cohort. Enhancers are defined as loci overlapping with H3K27ac and H3K4me1 while not overlapping with H3K4me3. CpGs mapped to TSS1500, TSS200, and the 5’ UTR in the EPIC array manifest are considered promoter. CpGs mapped to 1^st^ exon, body, and 3’ UTR in the EPIC array manifest are considered body.

**
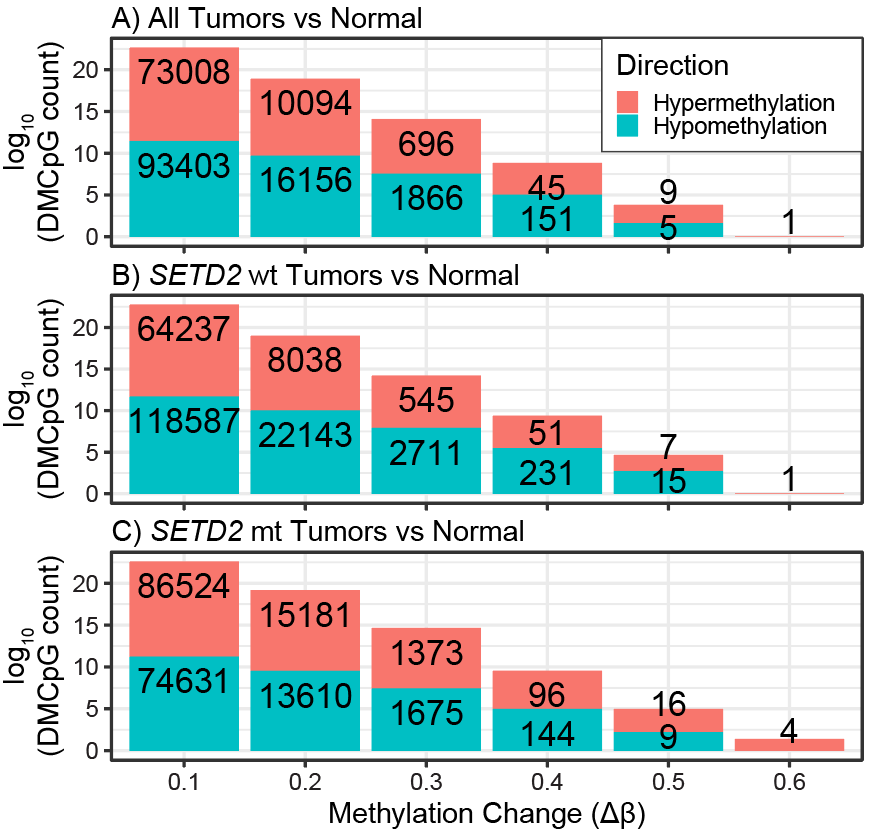
**

**Supplemental Figure S2.** DMCpGs at different Δβ cutoffs. **A)** Barplot of tumor versus normal kidney differentially methylated CpG (p < 0.01) counts. **B)** Barplot of *SETD2* wt tumors versus normal kidney differentially methylated CpG (p < 0.01) counts. **C)** Barplot of *SETD2* mt tumor versus normal differentially methylated CpG (p < 0.01) counts. Y-axis is the log_10_(DMCpG count).


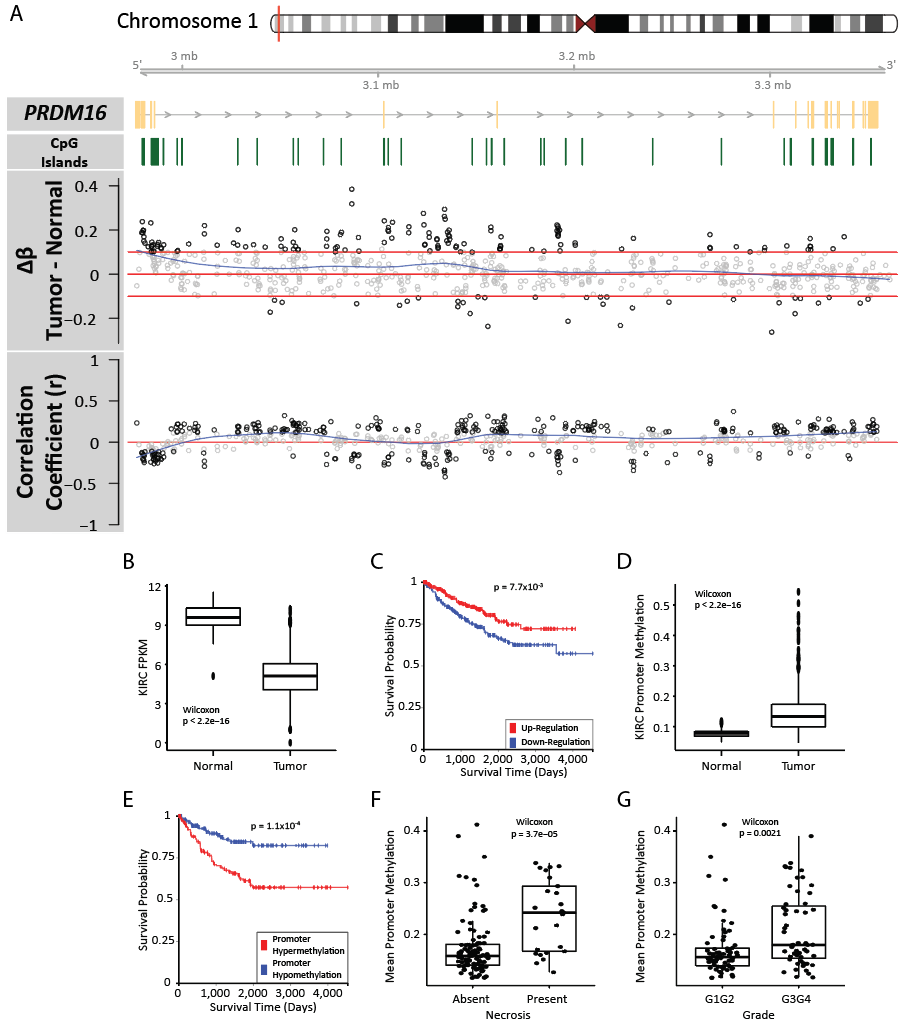


**Supplemental Figure S3.** **A)** Browser view of the *PRDM16* gene. Gene structure is displayed in the first track. Yellow bars represent exons and the grey line introns. Direction of transcription is indicated by arrows on the grey line. The second track displays CpG islands (green bars). Track 3 represent the Δβ_(Tumor- Normal)_ of all CpGs covered by the Illumina Methylation EPIC array and mapped to *PRDM16* using our cohort. Each grey circle represents a CpG, and the black circles represent the DMCpG called at p < 0.01 and |Δβ_(Tumor - Normal)_| > 0.1 cutoff. Red lines represent the cutoff line at Δβ_(Tumor- Normal)_ ±0.1. The blue line is a smoothed distribution of the data. Track 3 displays the correlation coefficient of CpGs mapped to *PRDM16,* in Illumina Methylation 450K array, and *PRDM16* gene expression in KIRC tumors. Each grey circle represents a CpG, and the black circles represent CpGs with a significant correlation (p < 0.05) with gene expression. The blue line is a smoothed distribution of the data. **B)** Boxplots showing *PRDM16* gene downregulation in KIRC tumor samples. **C)** Kaplan Meier curves showing better survival in ccRCC patients that have higher levels of *PRDM16* expression. **D)** Boxplot showing *PRDM16* gene promoter hypermethylation in KIRC tumor samples. **E)** Kaplan Meier curves showing better survival in patients with primary tumors showing *PRDM16* promoter hypomethylation*.* **F-G)** Boxplot showing *PRDM16* promoter hypermethylation in tumor regions containing necrosis or with high nuclear grade (G3/G4), in our cohort.


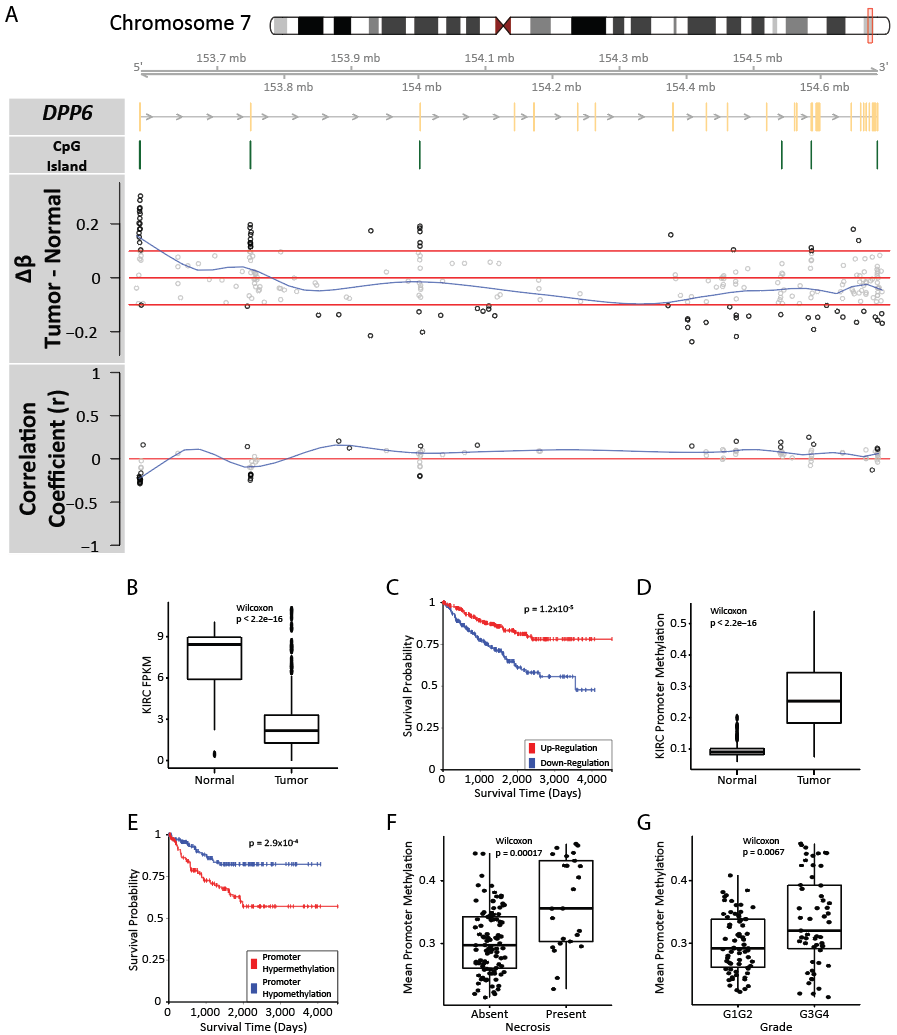


**Supplemental Figure S4.** **A)** Browser view of the *DPP6* gene. Gene structure is displayed in the first track. Yellow bars represent exons and the grey line introns. Direction of transcription is indicated by arrows on the grey line. The second track displays CpG islands (green bars). Track 3 represent the Δβ_(Tumor- Normal)_ of all CpGs covered by the Illumina Methylation EPIC array and mapped to *DPP6* using our cohort. Each grey circle represents a CpG, and the black circles represent the DMCpG called at p < 0.01 and |Δβ_(Tumor - Normal)_| > 0.1 cutoff. Red lines represent the cutoff line at Δβ_(Tumor- Normal)_ ±0.1. The blue line is a smoothed distribution of the data. Track 3 displays the correlation coefficient of CpGs mapped to *DPP6,* in Illumina Methylation 450K array, and DPP6 gene expression in KIRC tumors. Each grey circle represents a CpG, and the black circles represent CpGs with a significant correlation (p < 0.05) with gene expression. The blue line is a smoothed distribution of the data. **B)** Boxplots showing *DPP6* gene down regulation in KIRC tumor samples. **C)** Kaplan Meier curves showing better survival in ccRCC patients that have higher levels of *DPP6* expression. **D)** Boxplot showing *DPP6* gene promoter hypermethylation in KIRC tumor samples. **E)** Kaplan Meier curves showing better survival in patients with primary tumors showing *DPP6* promoter hypomethylation*.* **F-G)** Boxplot showing *DPP6* promoter hypermethylation in tumor regions containing necrosis or with high nuclear grade (G3/G4), in our cohort.


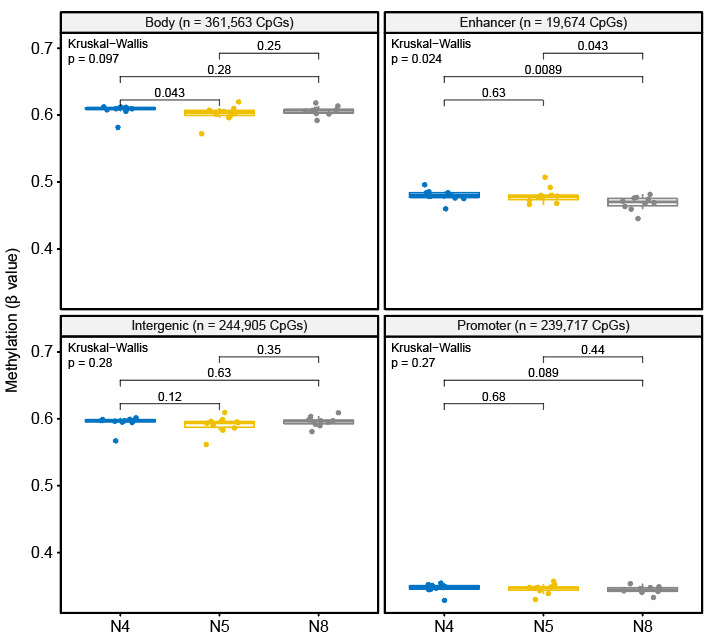


**Supplemental Figure S5.** Boxplots showing the feature-based DNA methylation levels for the regional analysis of three normal kidneys. Enhancers are defined as loci overlapping with H3K27ac and H3K4me1, but not H3K4me3. CpGs mapped to TSS1500, TSS200, and the 5’ UTR in the EPIC array manifest are considered promoter CpGs. CpGs mapped to 1^st^ exon, body, and the 3’ UTR in the EPIC array manifest are considered body CpGs.


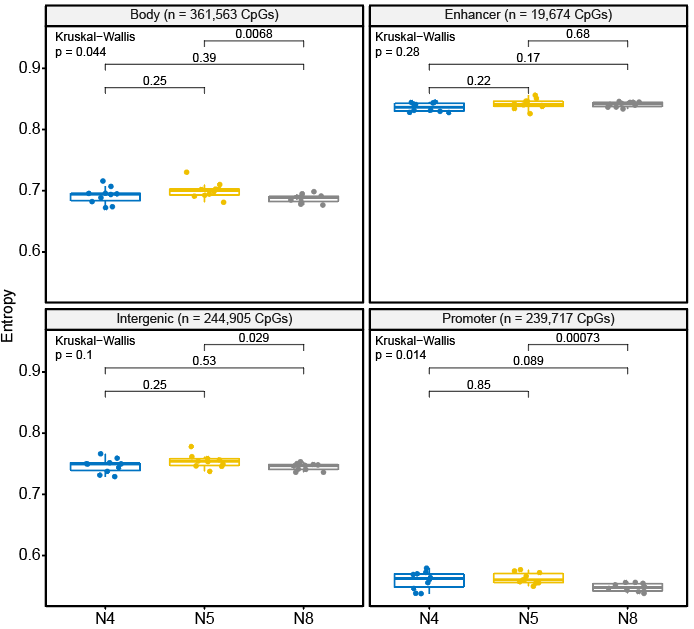


**Supplemental Figure S6.** Boxplots showing the feature-based entropy levels for the three normal kidneys. Enhancers are defined as loci overlapping with H3K27ac and H3K4me1 while not overlapping with H3K4me3. CpGs mapped to TSS1500, TSS200, and the 5’ UTR in the EPIC array manifest are considered promoter CpGs. CpGs mapped to 1^st^ exon, body, and the 3’ UTR are considered body CpGs.


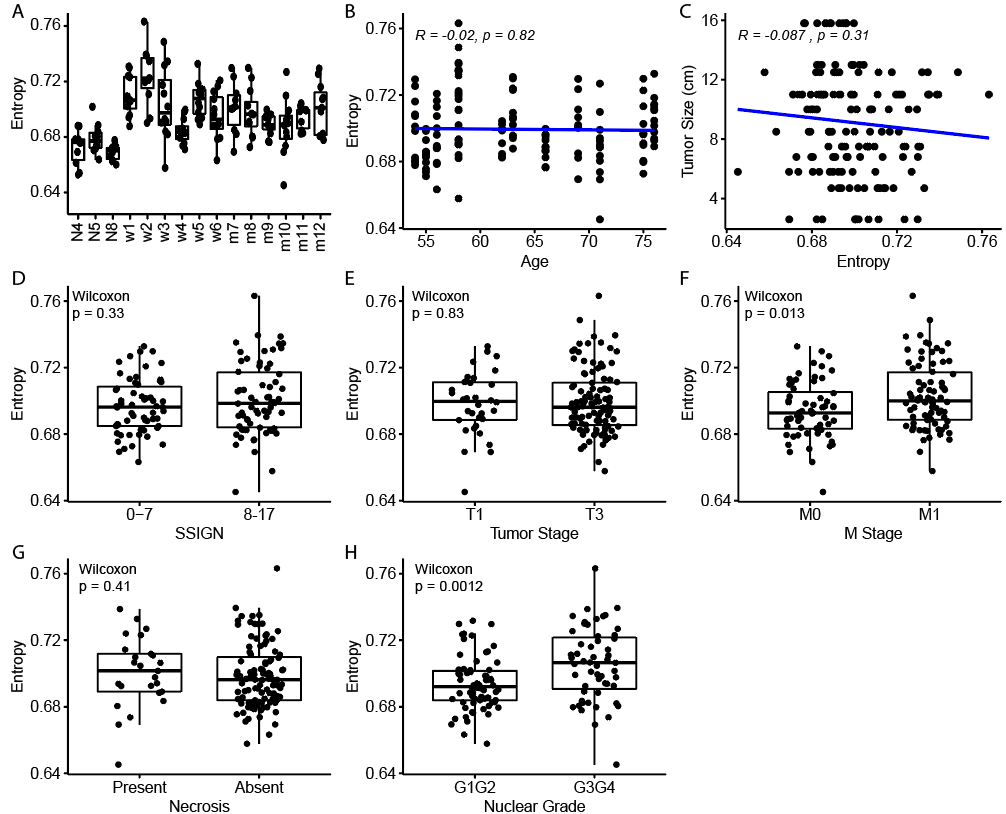


**Supplemental Figure S7.** CcRCC entropy calculated for all measured CpGs (n = 843,393). **A)** Boxplot showing entropy for each of the normal kidneys and the ccRCCs. Tumor samples have significantly higher entropy as they contain a larger amount of disorder. **B)** Scatterplot of the correlation calculation between chronological age of the ccRCC patients at time of surgery and entropy. **C)** Scatterplot of the correlation calculation between tumor size measured in centimeters (cm) and entropy. **D)** Boxplot showing no significant difference in entropy between low and high SSIGN score tumors. **E)** Boxplot showing no significant difference in entropy between tumors with low or high T stage. **F)** Boxplot showing a significantly higher entropy in ccRCCs (the primary tumor) that have metastasized (M1) relative to those that have not (M0). **G)** Boxplot showing no significant difference in entropy between tumor regions with or without necrosis. **H)** Boxplot showing a significantly higher entropy in ccRCC regions with higher nuclear grade.


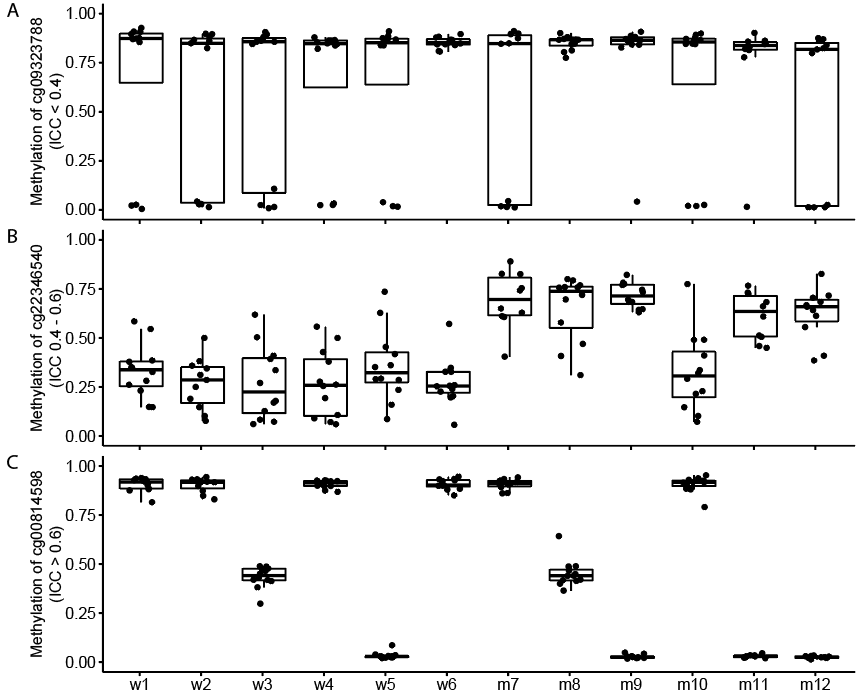


**Supplemental Figure S8.** Boxplot showing methylation of a single CpG in different regions of tumors. **A)** Methylation of cg09323788 (ICC < 0.4; low ICC) is highly variable within the same tumor. However, this variation is similar between tumors, leading to very similar means and medians. **B)** Methylation of cg22346540 (ICC 0.4 – 0.6; intermediate ICC) is less variable than that in panel A (boxplots are smaller). Here methylation is variable within each tumor while also being variable across tumors (differently sized boxplots). **C)** Methylation of cg00814598 (ICC > 0.6; high ICC) is not variable within the same tumor (small boxplot). However, the methylation is variable between tumors from different patients (different means and medians).


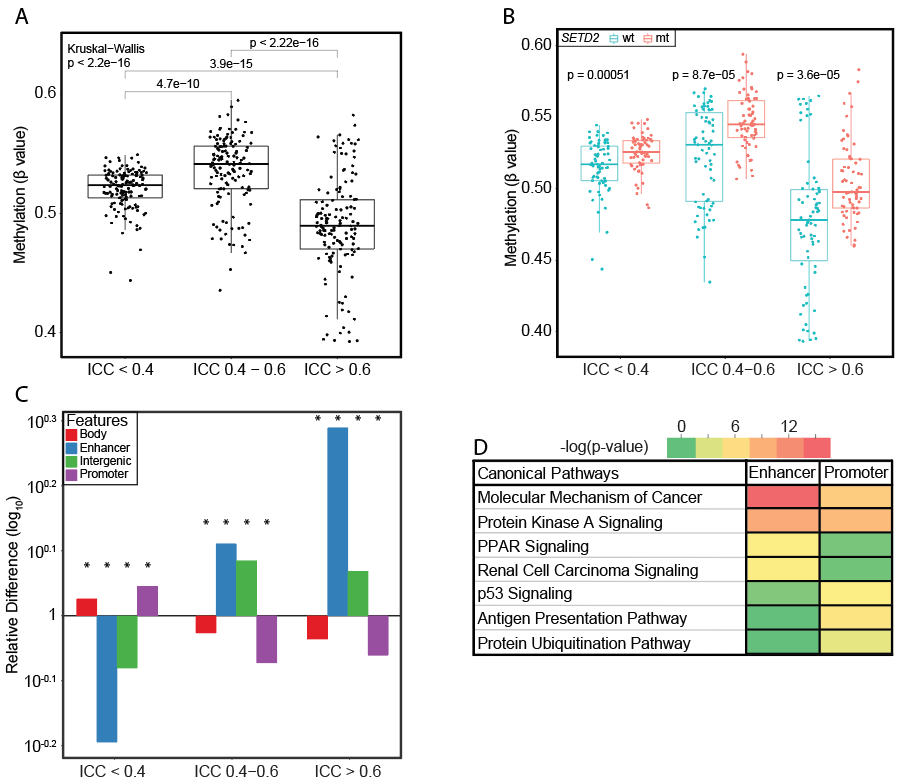


**Supplemental Figure S9.** Exploratory analysis of CpGs based on ICC grouping. **A)** Boxplot comparing global methylation of CpGs grouped according to ICC score calculated for all tumors. There are 138 points, representing all ccRCC tumor regions, scattered around each box. **B)** Boxplot comparing methylation of *SETD2* wt and mt tumors for the CpGs grouped by ICC score. **C)** Barplot showing the relative feature distribution of the CpGs in the EPIC array grouped by ICC score when normalized to the distribution of all CpGs on the array over four genomic features (intergenic, enhancer, promoter, and body). Enhancers are defined as loci overlapping H3K27ac and H3K4me1 but not H3K4me3. CpGs mapped to TSS1500, TSS200, and the 5’ UTR in the EPIC array manifest are considered promoter CpGs. CpGs mapped to 1^st^ exon, body, and the 3’ UTR are considered body CpGs. The asterisk indicates a significant distribution difference of the respective feature between the selected subset and the EPIC array. Y-axis – relative difference (log_10_) of each feature. **D)** Heatmap of ontology enrichments for a top group of pathways derived from 2,747 and 2,000 genes linked to the most variable enhancer and active promoter (defined as positive for H3Kme3 based on normal kidney) CpGs, respectively, from the ICC 0.4 – 0.6 group, using IPA. The color bar is representative of the strength of the association displayed as -log_10_(p-value).


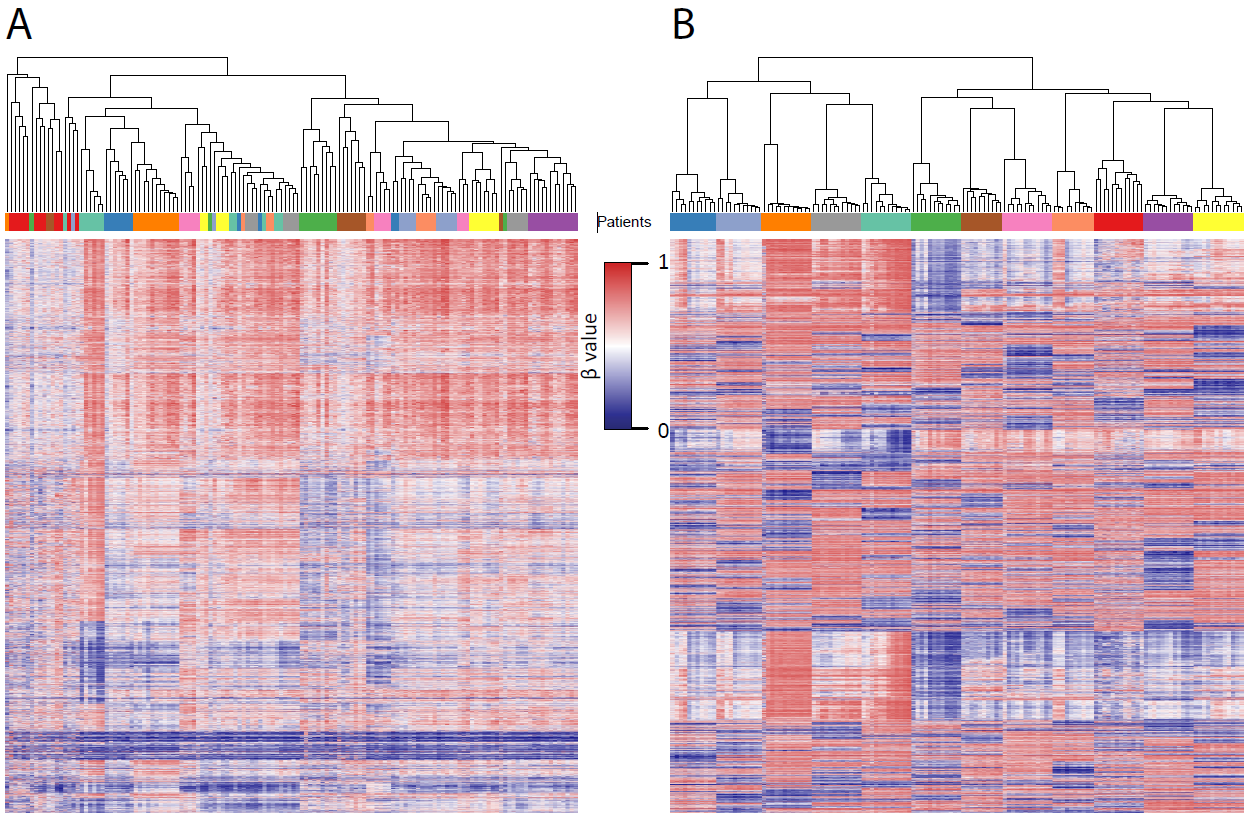


**Supplemental Figure S10. A)** Supervised hierarchical clustering using the 5,000 most variable CpGs from the low ICC group (ICC < 0.4). We observe CpGs with variation specific to their respective tumors and it is not possible to discern relevant methylation patterns (or distinct clusters). **B)** Supervised hierarchical clustering using the 5,000 most variable CpGs from the ICC > 0.6 group. We observe no separation of tumor regions. Instead, all regions are clustering based on the patient from which they originate. This group of CpGs highlight methylation variation between individual tumors/patients but not within. Coloring for patient ID is the same as in Fig. 1B.


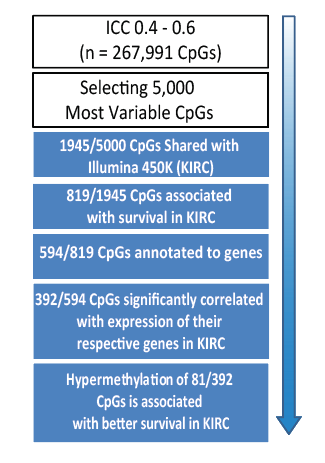


**Supplemental Figure S11**: Flowchart denoting relationships between the 267,991 CpGs in the ICC 0.4 - 0.6 group and survival in TCGA-KIRC. White boxes refer to information previously mentioned in Figs. 4G-H. Information specific for the analysis related to the KIRC dataset and survival is shown in the blue boxes. The progressive analysis of the overlapping CpGs for survival, and methylation-expression correlation are indicated in the flow diagram. This analysis was conducted using 318 KIRC ccRCCs for which both 450K and RNA-seq data are available.


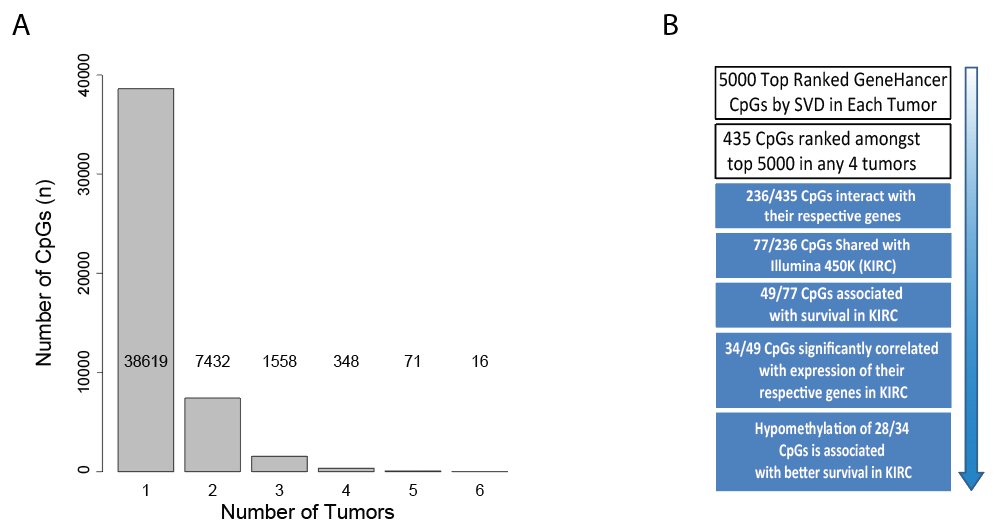


**Supplemental Figure S12**: **A)** Barplot showing the number of shared CpGs across any combination of tumors from our cohort. The x-axis corresponds to the number of tumors, the y-axis corresponds to the CpG count. The number of CpGs is indicated for each bar. **B)** Flowchart denoting relationships between the 5,000 highly ranked CpGs in each tumor by SVD and survival in TCGA-KIRC. White boxes refer to information derived from panel A. Information specific for the analysis of the CpGs we identified in the KIRC dataset, including survival relationships, is shown in the blue boxes. The successive steps taken to analyze the overlapping CpGs for survival, and methylation-expression correlation are indicated in the flow diagram. This analysis was conducted using 318 KIRC ccRCCs for which both 450K and RNA-seq data were available.


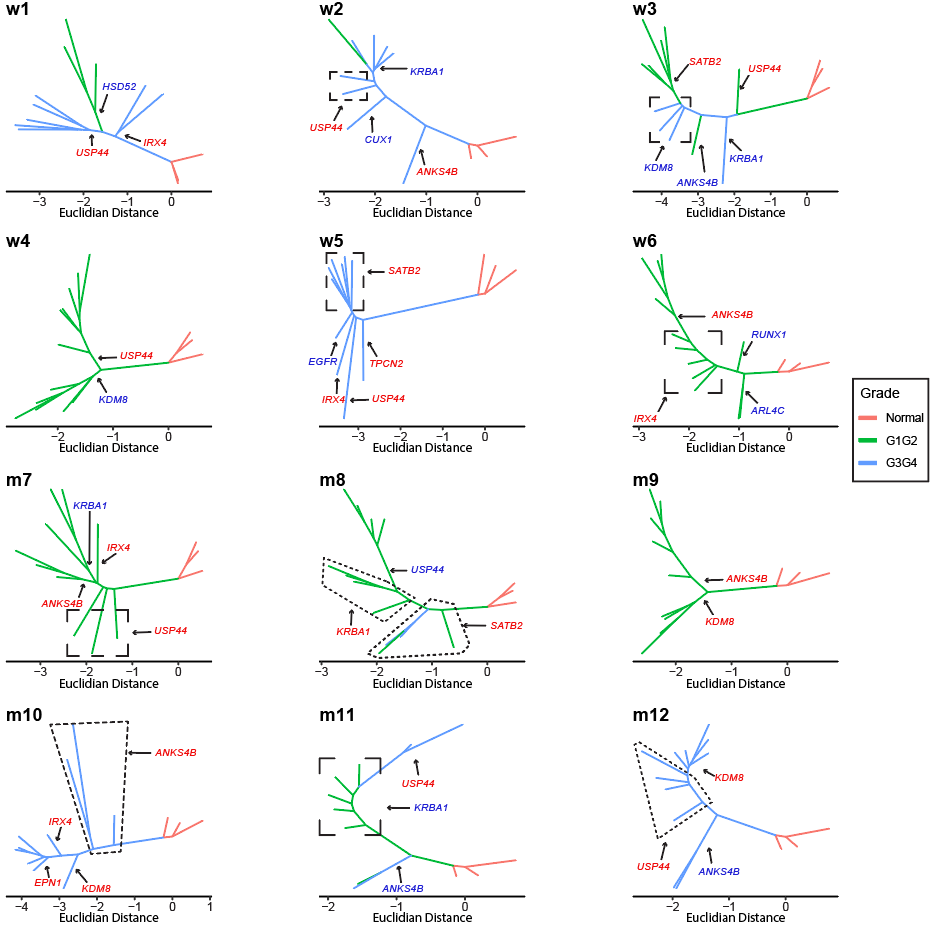


**Supplemental Figure S13**: Phyloepigenetic trees showing evolutionary methylation changes of regions from the same tumor for all 12 ccRCCs in our cohort. Branches represent tumor regions and are colored based on the nuclear grade scored from H&E slides. We highlight major evolutional intervals and identify ITH driver genes with the highest number of CpG methylation changes (red = hypermethylation; blue = hypomethylation).


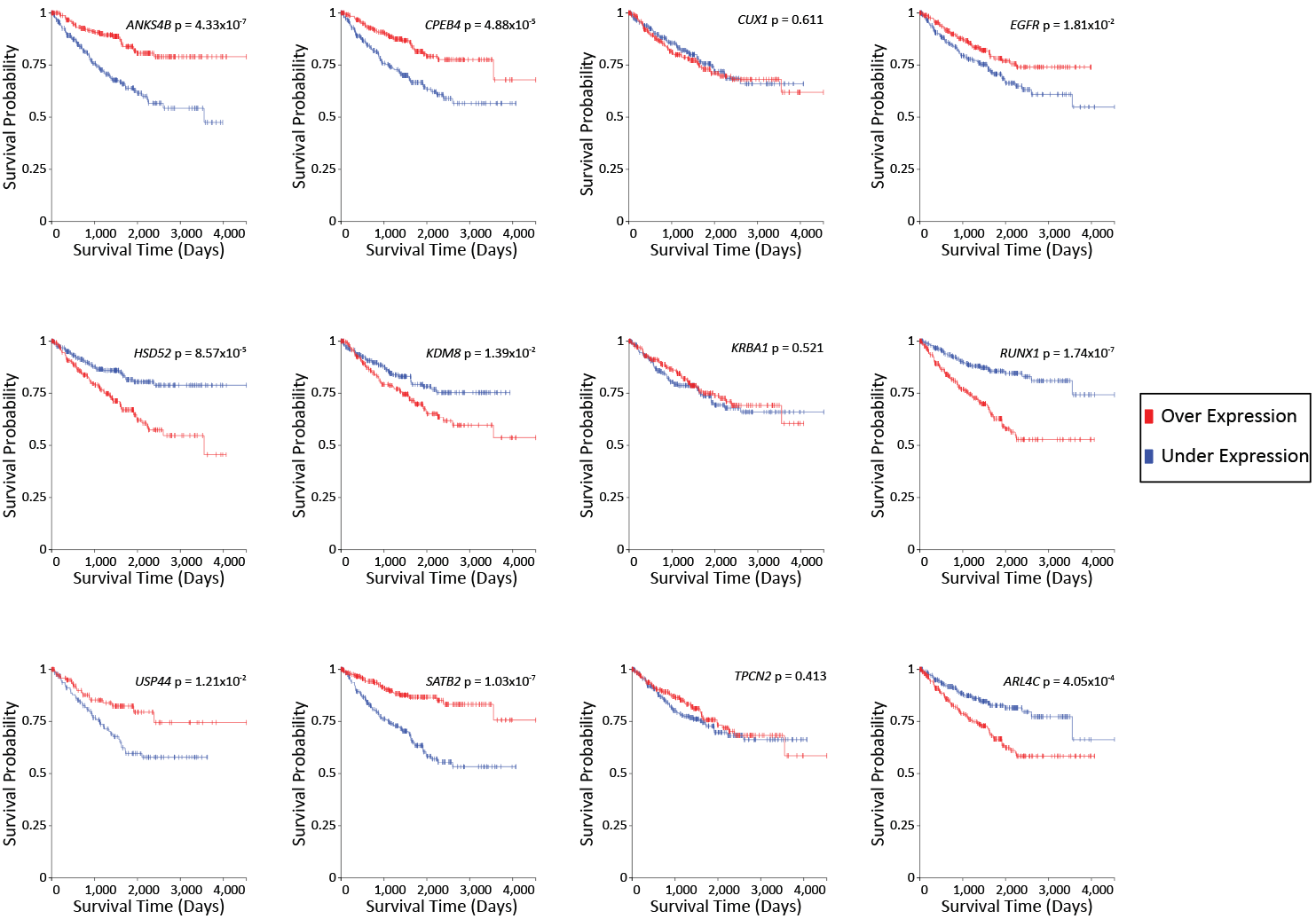


**Supplemental Figure S14**: Kaplan Meier curves contrasting survival outcomes between over-expression and under-expression of 12 genes noted in the phyloepigenetic trees from Supplemental Fig. S13. We note that survival data for *IRX4* is not available and therefore no Kaplan Meier curves could be generated for that gene.


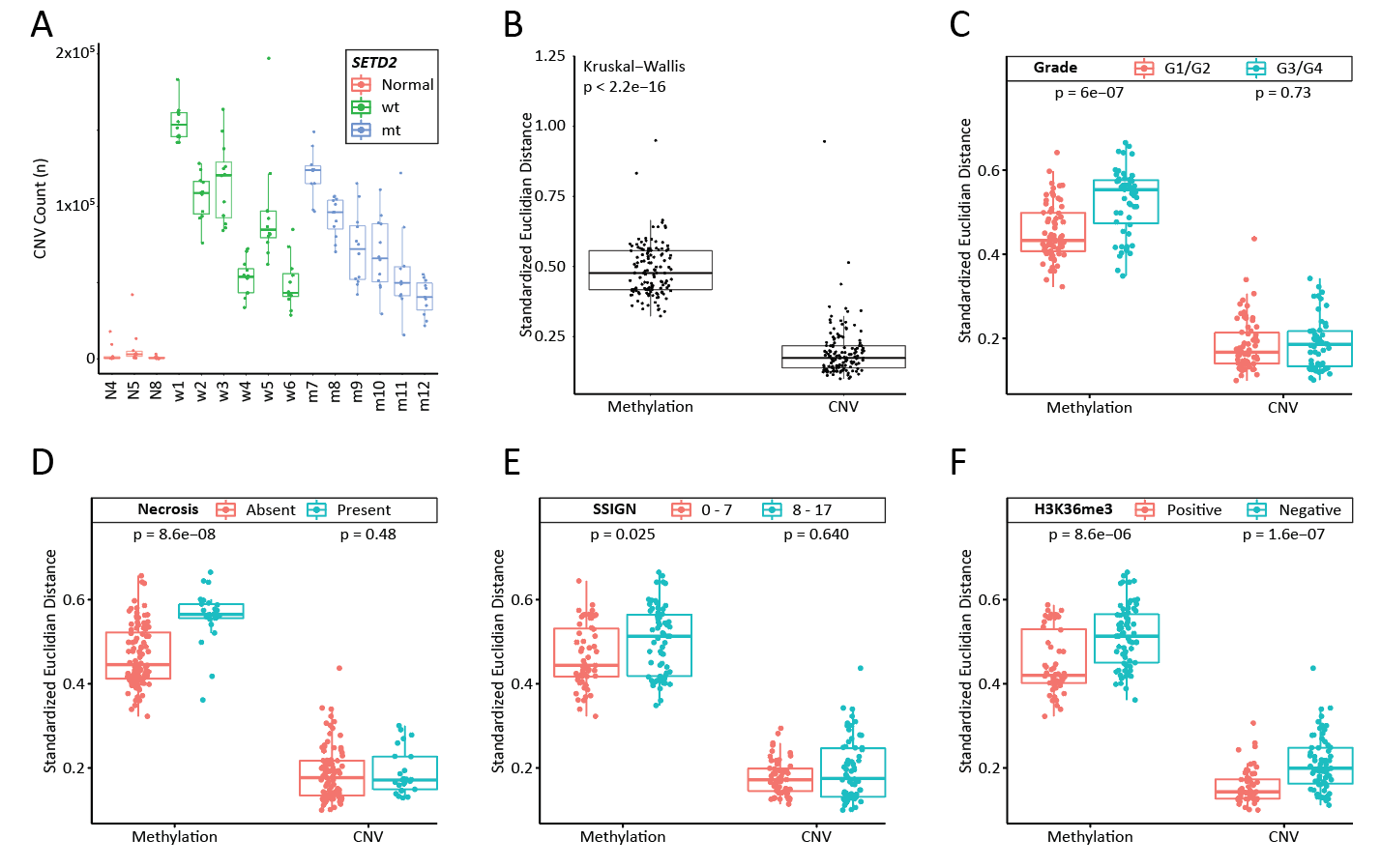


**Supplemental Figure S15**: ITH at the CNV level and its relationship with clinical and pathologic parameters. **A)** Boxplot of the CNV loci counts as determined by the *conumee* R package. The CNV count is the sum of loci with amplifications and deletions. Each dot represents a single tissue region. **B)** Boxplot showing a comparison of standardized Euclidian distance between the 5mC and CNV trees. Each point represents the mean distance between a tumor region and the normal samples in the phylo(epi)genetic trees in Fig. 6C. **C-F)** Boxplots showing a comparison of standardized Euclidian distance between the 5mC and CNV trees for four pathologic parameters. Points, boxes, and whiskers are colored according to nuclear grade **(C),** necrosis **(D)**, SSIGN score **(E)**, and H3K36me3 IHC **(F)**. Each point represents the mean distance between a tumor region and the normal kidney regions in the phylo(epi)genetic trees of Fig. 6C.


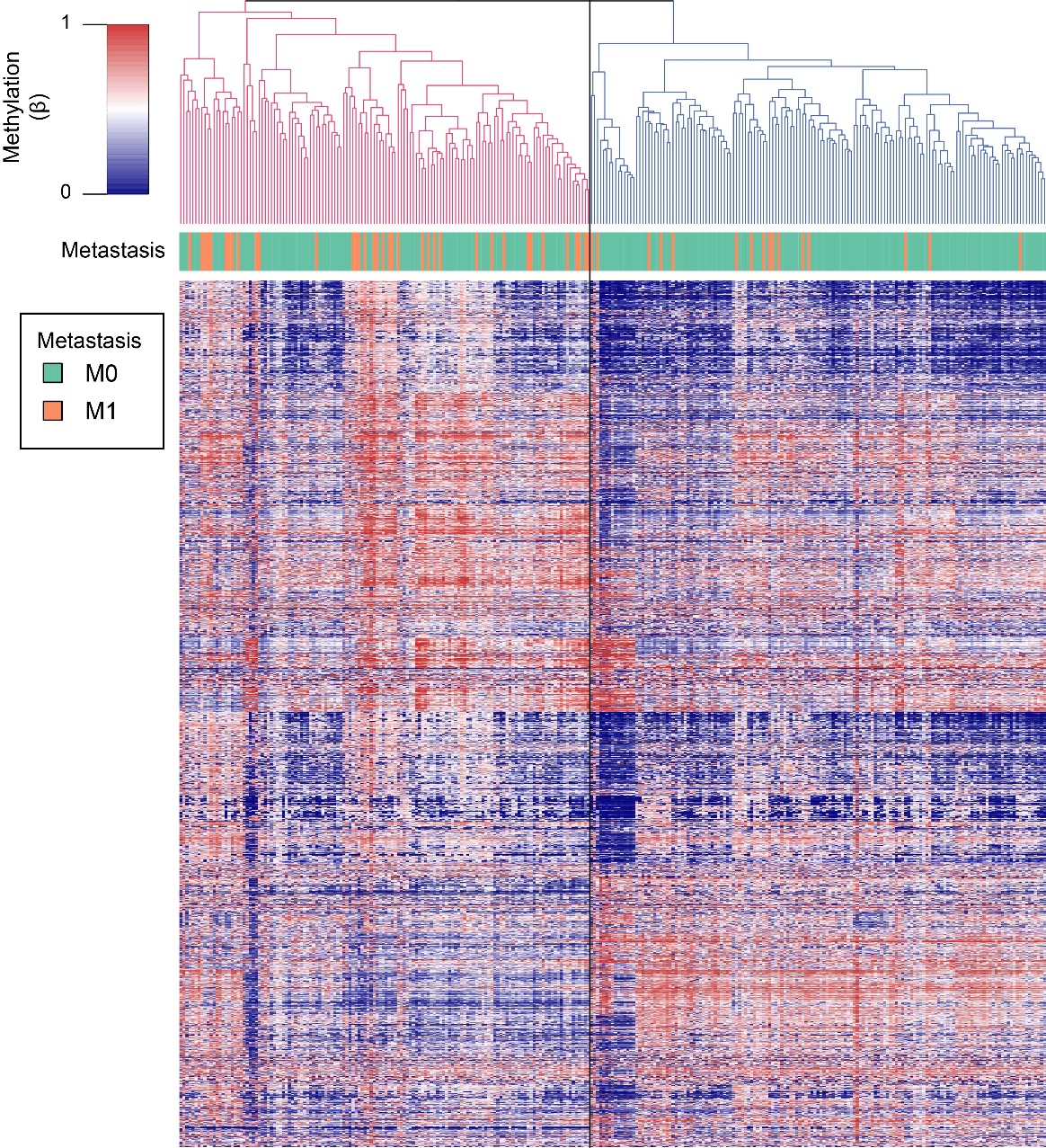


**Supplemental Figure S16:** Heatmap showing unsupervised hierarchical clustering using the 5,000 most variable CpGs in TCGA-KIRC samples with reported metastasis status (M0 = 234; M1 = 53). We observe the formation of two clusters with one (left) showing hypermethylation and an overrepresentation of ccRCCs that metastasized (M1 tumors, red branches, p = 5.23x10^-4^).


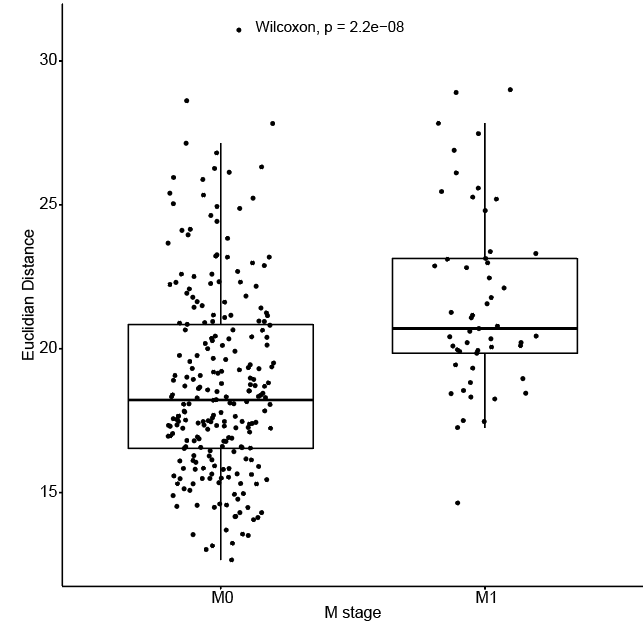


**Supplemental Figure S17:** Boxplot showing the Euclidian distance between M0 and M1 samples in KIRC and normal kidney samples adjacent to the KIRC tumors. Methylation data from KIRC is available for 234 M0 primary tumors, and 53 M1 primary tumors.
